# Review on Computer-Aided Design and Manufacturing of Drug Delivery Scaffolds for Cell Guidance and Tissue Regeneration

**DOI:** 10.3389/fbioe.2021.682133

**Published:** 2021-06-24

**Authors:** Aurelio Salerno, Paolo A. Netti

**Affiliations:** ^1^Independent Researcher, Barcelona, Spain; ^2^Center for Advanced Biomaterials for Healthcare, Istituto Italiano di Tecnologia, Naples, Italy; ^3^Department of Chemical, Materials and Industrial Production Engineering, University of Naples Federico II, Naples, Italy; ^4^Interdisciplinary Research Center on Biomaterials, University of Naples Federico II, Naples, Italy

**Keywords:** additive manufacturing, biomimetic scaffolds, computer-aided design (CAD) processes, drug delivery, growth factor

## Abstract

In the last decade, additive manufacturing (AM) processes have updated the fields of biomaterials science and drug delivery as they promise to realize bioengineered multifunctional devices and implantable tissue engineering (TE) scaffolds virtually designed by using computer-aided design (CAD) models. However, the current technological gap between virtual scaffold design and practical AM processes makes it still challenging to realize scaffolds capable of encoding all structural and cell regulatory functions of the native extracellular matrix (ECM) of health and diseased tissues. Indeed, engineering porous scaffolds capable of sequestering and presenting even a complex array of biochemical and biophysical signals in a time- and space-regulated manner, require advanced automated platforms suitable of processing simultaneously biomaterials, cells, and biomolecules at nanometric-size scale. The aim of this work was to review the recent scientific literature about AM fabrication of drug delivery scaffolds for TE. This review focused on bioactive molecule loading into three-dimensional (3D) porous scaffolds, and their release effects on cell fate and tissue growth. We reviewed CAD-based strategies, such as bioprinting, to achieve passive and stimuli-responsive drug delivery scaffolds for TE and cancer precision medicine. Finally, we describe the authors’ perspective regarding the next generation of CAD techniques and the advantages of AM, microfluidic, and soft lithography integration for enhancing 3D porous scaffold bioactivation toward functional bioengineered tissues and organs.

## Introduction to Computer-Aided Design and Manufacturing of Drug Delivery Scaffolds

Advanced drug therapies require customization and targeting of drug formulation and dosage to each specific patient to warrant treatment efficacy and reduce possible undesired secondary effects. To this purpose, engineering strategies for drug delivery system design and fabrication necessitate the combination and manipulation of materials and drugs to obtain even complex bioactive systems. Most specifically, the composition, chemical functions, morphology, and architectural features of new drug delivery systems must be controlled and designed at nanometric-scale resolution.

**FIGURE 1:**
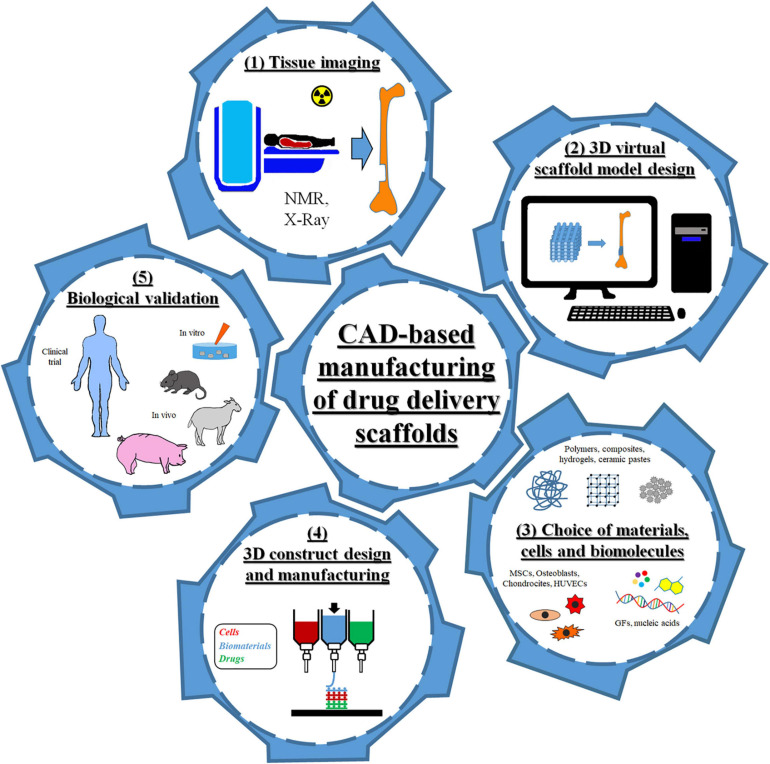
Scheme of the different steps of computer-aided design (CAD)-based approaches for the fabrication of drug delivery scaffolds for tissue engineering.

In the past decade, the combination of computer-aided design (CAD) and additive manufacturing (AM) has revolutionized the fields of personalized medicine and drug delivery systems (Guzzi and Tibbitt, 2020; Mohammed et al., 2020). Indeed, CAD-AM approaches have enabled the manufacturing of biomedical devices with unique features for *in vitro* and *in vivo* applications. Some examples are three dimensional (3D) drug delivery scaffolds for tissue growth and repair as well as 3D models for cancer precision medicine (Moreno Madrid et al., 2019; Shafiee, 2020). As shown in Figure 1, this broad category of design and fabrication techniques used medical imaging combined with virtual scaffold models and automated layer-by-layer processing to produce patient-specific devices characterized by highly controlled geometrical features, reliable microstructural properties, and spatial and temporal drug delivery capability. In particular, data acquired from computerized tomography or nuclear magnetic resonance (NMR) tests were used to generate a customized CAD model and define the consequent scaffold geometry and internal features to fit the specific tissue defect site. The scaffold model was subsequently divided into multiple layers for fabrication. AM techniques are modular approaches based on the assembly/sintering of layered structures obtained by continuous or discontinuous processes (Salerno et al., 2019). The advantages of employing AM processes, such as 3D printing, include the capability of precisely controlling the spatial loading of an active molecule within even minute quantities and generate multiple delivery profiles by creating different depots and complex geometries (Caballero-Aguilar et al., 2020; Jacob et al., 2020). These aspects enabled the compounding of personalized dosage form to minimize costs, to improve patient compliance, and to maximize drug efficacy. Besides, 3D printing technology can be successfully used in initial stages of drug development and testing, including preclinical studies and trials of dosage form with excellent dose flexibility (Jacob et al., 2020). The quality of the produced device can be adjusted by altering the fabrication parameters, mainly printing inkjets and speed, substrate of deposition, and extrusion parameters (e.g., temperature and pumping pressure) (Datta et al., 2018; Parak et al., 2019; Wang et al., 2020).

The aim of this work is to review the recent advances of CAD-AM processes focusing on the preparation of even complex drug delivery scaffolds for cell guidance and tissue repair. In particular, emphasis will be devoted to those processes/approaches allowing the fabrication of multifunctional extracellular matrix (ECM)-mimicking scaffolds and stimuli-responsive drug-loaded devices for tissue engineering (TE) and cancer precision medicine. Insight into current drawbacks and future challenges of CAD-AM processes are also provided in the concluding section of this work.

## Strategies for Porous Scaffold Bioactivation by Drug Entrapment and Delivery

Tissue engineering aims to repair and restore damaged tissue functions by using ECM-mimicking drug-releasing scaffolds or by incorporating drug delivery devices into TE scaffolds (Salerno et al., 2017; Calori et al., 2020). The ECM is a hierarchical biomolecular environment in which many cell-signaling molecules are continuously synthesized, sequestered, and released aiming to modulate cell adhesion, maintenance and self-renewal, and to guide cell proliferation, migration, and differentiation behaviors (Dutta and Dutta, 2009). For instance, the ECM of soft connective tissues is composed of fiber-forming proteins, such as collagens, elastin, and fibronectin organized into collagenous nanofibrous bundles (Ghosh et al., 2019). Furthermore, a glycosaminoglycan and proteoglycan hydrogel fills the pores of this woven fibrous bundle. These polysaccharides contain numerous instructive signals and soluble factors secreted by the resident cells that are critical for tissue development, homeostasis, and repair, and that influence cell-mediated assembly and degradation of ECM components (Ghosh et al., 2019). Besides, the natural cellular environment is heterogeneous and dynamic as the ECM composition and structure change with tissue site and developmental stage (Peng et al., 2021).

In the TE field, 3D porous scaffolds are central elements for tissue regeneration *in vitro* and/or *in vivo* as they regulate essential cellular events such as adhesion, migration, proliferation, and morphogenesis (Salerno et al., 2013, 2014; Bruggeman et al., 2017). Furthermore, scaffolds must encode arrays of biological signals, with an adequate dose and for a desired period, to cell-surface receptors to recapitulate the spatial and temporal microenvironments presented by the natural ECM. Growth factors (GFs) are biomolecules belonging to a family of intracellular signaling polypeptides able to modulate cellular activities, such as stimulating or inhibiting cellular proliferation, induce stem cell migration and recruitment from adjacent tissues, and direct their differentiation (Bittner et al., 2018). Naturally, GF stimuli are transmitted into the cell via activation of specific, transmembrane receptors that influence important regulatory proteins residing into the cytoplasm. These proteins, in turn, control cellular activities, including changes in gene expression and response to other factors (Cross and Dexter, 1991). The responding cell type, concentration of factor, and presence of other stimuli, often in a complex variable manner, determine GF effect (Wang et al., 2009). In TE strategies, GFs can be supplied directly into the culture medium at regular intervals to guide cell behavior *in vitro*. However, direct administration *in vivo* is difficult, as it requires large delivery quantities to overcome possible GF inactivation and clearance. High GF levels are, in fact, associated with high risk of adverse effects and increasing treatment costs (Wang et al., 2009). GF encapsulation strategies allowed researchers to overcome these limitations as the encapsulating material protects these molecules, while their delivery can be controlled by the modulation of carrier composition, size, and structure (Calori et al., 2020; Hwa Kim et al., 2020). Although GFs are among the most used biomolecules in TE, scaffolds delivering genetic material, including DNA and RNA, may provide a potential alternative to GFs as nucleic acids can induce changes in the gene expression of cells (Biondi et al., 2008; Kelly et al., 2019). For example, transplanted cells can take up the delivered DNA and be transfected to express proteins that may aid in healing a defect. As DNA aims to encode for new protein production, it must first enter the cell and then reach the nucleus often by the aid of viral vectors (Kelly et al., 2019). Antibiotic, anti-inflammatory, or differentiation agents are other drugs that can be useful for TE purposes (Wang et al., 2014). Implantation of engineered scaffolds might, in fact, cause local prolonged inflammation owing to the host immune response, which therefore requires the use of anti-inflammatory agents (Li et al., 2018). Glucocorticoids (e.g., dexamethasone) or non-steroids (e.g., ibuprofen) were delivered by the implanted biomaterials to control and modulate the local inflammatory response, avoiding possible side effects associated with systemic administration (Cantón et al., 2010; Li et al., 2018). Similarly, scaffolds delivering antibiotics, such as gentamicin, vancomycin, and antibacterial ions, may prevent infections from occurring after implantation (Yao et al., 2013; Visscher et al., 2018). It is therefore clear that the loading, as well as spatial and temporal delivery of bioactive factors from porous scaffolds is an important issue of scaffold bioactivation, and it will be discussed in the next paragraphs.

### Biomolecule Delivery by Passive Release

One of the most used strategies for bioactive factor loading into scaffolds relies on the physical entrapment of signaling molecules within the scaffold matrix. This approach is widely adopted for scaffolds made of hydrogels, as biomolecules can be easily and safely loaded into the polymeric solution mixture before crosslinking or, alternatively, by swelling crosslinked samples into a solution containing the biomolecules. The delivery of the loaded factors is often a balance between free diffusion and hydrolytic degradation of the polymeric material, and can be tuned by choosing the properties of the entangled fiber structure, such as surface area, pore size, and mesh size (Li and Mooney, 2016; He et al., 2020; Shultz and Zhong, 2021). In particular, when the hydrodynamic diameter of the diffusing molecule approaches the hydrogel mesh size, the release is not only dependent on diffusion but also is controlled by polymer degradation, either hydrolytic or enzymatic. As a direct consequence, the delivery rate from hydrogel scaffolds is lowered by increasing crosslink density and polymer concentration (He et al., 2020). Both synthetic and natural polymers have been used for the design of hydrolytically degradable hydrogels in which chemical or physical crosslinking offers the possibility of controlling the diffusion of solubilized hydrophilic drugs. Naturally derived hydrogels, such as collagens, hyaluronic acid, and derivatives, are excellent materials for hydrogel preparation due to their chemical composition and structure resembling the features of the native ECM (Skardal et al., 2017; Mondal et al., 2020). For example, Skardal et al. (2017) optimized a fast photocrosslinkable heparin-conjugated hyaluronic acid hydrogel system capable of sequestering and releasing growth factors secreted from encapsulated cells. Furthermore, the authors varied hydrogel crosslinking to obtain a sustained release of proteins and heparin-binding growth factors (Skardal et al., 2017). The breakdown of polymeric chains, by hydrolysis or enzyme activity, causes the hydrogel structure to rupture and accelerates the release of drugs (Campbell et al., 2018). Hydrogel features that affect water diffusion, such as pore size and crosslink density, can also have a direct role on polymeric chain degradation and, therefore, modulate hydrogel degradation rate. Wang et al. (2018) designed an injectable macroporous hydrogel composed of gelatin/oxidized alginate/adipic acid dihydrazide loaded with human epidermal growth factor for self-healing purposes. The obtained hydrogels had an interconnected macroporous structure with porosity in the 60%–83% range and pore size from 125 to 380 μm. The authors observed that increasing hydrogel pore size and porosity accelerated the degradation and resulted in a faster growth factor release. Similarly, Campbell et al. (2018) designed an injectable alginate hydrogel that becomes porous *in situ* to enhance vascular progenitor cell release. The group of Ehrbar et al. (2007) also demonstrated the importance of hydrogel composition and degradation on biomolecule release and, therefore, tissue regeneration. In their work, biomolecular poly(ethylene glycol)-based hydrogels synthesized and degraded via site-specific enzymatic reactions were developed. These hydrogels evidenced cell-secreted metalloproteinase degradation properties mimicking the cell material crosstalk occurring in the native ECM. By this way, the authors engineered novel scaffolds for the cell-mediated modulation of biomolecule release (Ehrbar et al., 2007).

Vascular endothelial growth factor (VEGF)-delivering hydrogels have been widely used to enhance cell survival and scaffold vascularization in 3D. Indeed, VEGF initiates the sprouting of existing blood vessels by its mitogenic and chemotactic effects, drives the processes of angiogenesis and arteriogenesis, and stimulates the rapid development of a vascular network within 3D scaffolds (Cao and Mooney, 2007). However, VEGF efficacy is dose-dependent as downregulation can be unsuccessful at stimulating blood vessel-forming processes, while upregulation can produce an uncontrollable and detrimental blood vessel growth. VEGF-loaded alginate hydrogels have been deeply tested by Sun et al. (2005) and Cao and Mooney (2007) to validate the efficacy of VEGF release matrixes in the treatment of ischemic tissue. Alginate hydrogels were chosen as delivery scaffolds because VEGF can be easily loaded into the hydrogel at desired concentration and without significant growth factor deactivation during manufacturing. Concomitantly, the hydrogel provided a controlled release into the local cellular microenvironment to yield desirable concentrations over a period of days to months (Cao and Mooney, 2007; Shvartsman et al., 2014). Although VEGF is a well-established initiator of angiogenesis, its presence is often not sufficient for the formation of a complex, mature vascular network, and it was necessary to deliver multiple morphogens acting in distinct aspects of the tissue regeneration process to drive tissue regeneration to completion. Drug delivery hydrogel strategies combined VEGF with insulin-like growth factor-1 to promote functional innervation (Borselli et al., 2010a; Raimondo et al., 2019). Alternatively, VEGF and platelet-derived growth factor (PDGF) were used to stimulate blood vessel maturation and stabilization by muscle cell recruitment (Hao et al., 2007), while VEGF and bone morphogenetic protein-2 (BMP-2) enhanced osteogenic and vasculogenic differentiation of hydrogel-encapsulated cells for bone regeneration (Barati et al., 2016).

Synthetic solid biodegradable materials were also used in TE to prepare drug delivery platforms, especially for load-bearing applications. This is because, different from hydrogels, scaffolds made of these materials have mechanical properties suitable for hard-tissue repair (Lin et al., 2020). However, growth factor encapsulation within solid scaffolds posed serious issues regarding bioactive molecule leaching and degradation during processing. Reducing the use of organic solvents and/or high temperatures during the manufacturing processes and avoiding contact between the protein and aqueous solution are consequently key issues to protect biomolecule functionalities (Borselli et al., 2010b). The most currently used bio-safe technology for the production of drug-loaded devices is supercritical CO_2_ (scCO_2_) technology as it allows for upscaling drug deactivation problems related to the use of organic solvents and/or high temperatures (Salerno et al., 2015). Indeed, CO_2_ is ecofriendly and non-flammable, whereas scCO_2_ is achievable at a rather low critical temperature (Tc = 31.1°C) and moderate critical pressure (Pc = 7.4 MPa) (Salerno et al., 2017, 2018). Several works reported the use of scCO_2_ as a blowing agent for thermoplastic biocompatible polymer foaming and porous scaffold manufacturing (Champeau et al., 2015; Salerno and Domingo, 2015). For example, porous scaffolds made of VEGF-loaded polylactic-co-glycolic acid (PLGA) were prepared by a high-pressure CO_2_ fabrication process (Sun et al., 2005). Briefly, PLGA microspheres were mixed with human VEGF lyophilized with alginate and salt particles, and the mixture was processed with CO_2_ at 5.5-MPa pressure and room temperature for 72 h. When the pressure was released, the PLGA particles expanded into the spaces between the salt particles and fused, trapping VEGF and the salt. Subsequently, the salt particles were leached out in water to yield porous scaffolds. The as-prepared porous scaffolds evidenced sustained VEGF delivery for up to 2 months and were able to promote *in vivo* tissue perfusion, greater capillary density, and more mature vasculature if compared with the VEGF-free PLGA scaffold used as control (Sun et al., 2005). More recently, de Rieux et al. (2011) enhanced VEGF loading efficiency into gas foaming/salt leaching porous scaffolds by growth factor loading into chitosan nanoparticles before scaffold incorporation. In fact, the addition of GF-encapsulating carriers within porous scaffolds is a suitable way to enhance bioactive factor loading and delivery. Using nano- and micro-carrier delivery systems also opens new routes for the engineering of scaffolds releasing multiple GFs. Richardson et al. (2001), to direct the formation of a mature vasculature, tested the dual delivery of VEGF and PDGF from porous PLGA scaffolds. PDGF was pre-encapsulated in PLGA microspheres by double emulsion, while VEGF was incorporated into the PLGA scaffold matrix by CO_2_ foaming. The fast VEGF delivery induced the rapid initiation of blood vessels, while the late PDGF delivery from PLGA microspheres promoted the stabilization of the preformed vascular network, finally demonstrating the versatility of this approach to study blood vessel regression and remodeling upon controlled GF release (Richardson et al., 2001). Microsphere-loaded porous scaffolds prepared by gas foaming/salt leaching were also used for bone regeneration. In particular, PLGA microspheres loaded with either VEGF and BMP-2 were incorporated into PLGA porous scaffolds to evaluate the *in vivo* osteogenic response to different GF ratios (Hernández et al., 2012).

### Scaffold Bioactivation by Physical–Chemical-Triggered Biomolecule Release

Many applications in medicine require controlled release devices able to provide a pulsed protein and peptide release profile. This is the case, for example, in hormone and vaccine release, for drugs with an extensive first-pass metabolism and that develop biological tolerance when they are constantly present at their target site, and for drugs that require administration during sleeping (Stubbe et al., 2004). Adaptable drug delivery biomaterials represent the cutting edge of biomedical engineering, as drug delivery can be “programmed” by the inner mechanism of the device (e.g., degradation) or “triggered,” where the release is governed by changes in the physiologic environment. Temperature-responsive hydrogels, made of lower critical solution temperature (LCST) polymers, are liquid below a critical solution temperature and become a gel above it. Physiological gelation temperatures enable injectable materials, such as poly(*N*-isopropylacrylamide) (PNIPAAm) and chitosan-based solutions, to be administered through a syringe and gel upon injection into the body, where they may serve as a drug or biomolecule reservoir (Pal et al., 2020; Tao et al., 2020). PNIPAAm features hydrophilic amide groups, which are buried during its coil-to-globule transition above the LCST point, and hydrophobic isopropyl groups, which are conversely exposed. On the contrary, chitosan is not inherently thermoresponsive, while the addition of phosphate salts, polyol-phosphates, and polyol molecules yields a thermogelling system with an LCST in the 15–85°C range. Chitosan and PNIPAAm can be also combined to form multiphase wound healing hydrogels where chitosan imparted improved biocompatibility, while PNIPAAm provided a thermally triggered volume change for enhanced control of drug delivery (Hogan and Mikos, 2020). Another approach in developing “smart’ multiresponsive hydrogels is via the incorporation of temperature-sensitive additives, such as liposomes or nanoparticles (Lu and Ten Hagen, 2020; Palmese et al., 2020). Recently, Pedersen et al. (2020) developed hydrogel biomaterials with triggered liquefaction in response to internal, localized heating, mediated by near-infrared light as external stimulus. This adaptable behavior was obtained by combining poly(vinyl alcohol) hydrogel with gold nanoparticles or an organic photothermal dye as heat generators. Upon laser light irradiation, composite hydrogel underwent liquefaction within seconds allowing the controlled, on-demand release of the incorporated cargo (Pedersen et al., 2020).

Thermoresponsive polymeric nanocarriers, including micelles, liposomes, dendrimers, and polymersomes are other interesting systems for drug delivery purposes. Liposomes that are characterized by an aqueous core surrounded by one or more concentric lipid bilayer allowed loading of either hydrophilic or hydrophobic drug molecules, while their release behavior was engineered to respond to external stimuli such as heat, light, ultrasound, and pH. Thermosensitive liposomes (TSLs) are among the most studied due to their ability to generate rapid and massive drug release in the heated area, and marginal release of contents in non-heated parts of the body (Lu and Ten Hagen, 2020; Yuba, 2020). This rapid release feature of TSLs occurred at a temperature range at which the liposomal membrane is going through a phase transition, which causes membrane openings and drug release. During the phase transition, manipulating temperatures can alter the density of gaps in the liposomal membrane, thus, also controlling the amount of released biomolecules. Typically, the temperature range for clinical hyperthermia is 40–45°C. Therefore, temperature-responsive liposomes that can show sharp responsiveness at this temperature range are promising in a viewpoint of clinical application. Clinically, liposome-based delivery systems were used for the delivery of bioactives, such as genes, drugs, and other biological molecules, especially for applications such as cancer treatment. Zhang et al. (2014) developed docetaxel-encapsulated thermosensitive liposomes for the targeted delivery of a drug to a tumor. The release rate of DOX was high at 42°C compared with 37°C and enabled higher tumor growth suppression *in vivo* if compared with the free drug-treated group. Growth factor receptor-bound protein-2 liposomes were prepared to inhibit the production of the growth factor receptor-bound protein-2 and, thereby, to reduce the proliferation of tumor cells (Saraf et al., 2020). TSL administration can be done directly in suspension (e.g., intravenous injection) or by loading them into injectable hydrogels to sequential delivery of multiple drugs (Lu and Ten Hagen, 2020; Palmese et al., 2020). In a recent work, Palmese et al. (2020) synthesized an injectable crosslinked poly(ethylene glycol) hydrogel containing both chemically crosslinked TSLs and matrix metalloproteinase-sensitive peptide crosslinks capable of independently responding to matrix metalloproteinase and applied hyperthermia. Doxorubicin, a widely used anticancer drug, was loaded in the TSLs with a high encapsulation efficiency, and the subsequent release was temperature dependent. Experiments characterizing the *in situ* drug delivery and degradation of these materials indicate that the TSL gel responds to both thermal and enzymatic stimuli in a local environment. The timescales of release associated with these two stimuli are distinct, allowing for the potential loading and independent delivery of multiple compounds (Palmese et al., 2020).

Light as an external stimulus for smart drug delivery systems is advantageous for a number of reasons including its non-invasive nature, high spatial resolution and temporal control, and convenience and ease of use. Light-based strategies used to design novel delivery systems can be classified into three main groups (Linsley and Wu, 2017; Ruskowitz and DeForest, 2018): photochemically triggered, where the absorbed light energy is sufficient to break covalent bonds directly or by a photochemical reaction; photoisomerization, where the excess energy causes structural changes; and photothermal, where the absorbed photon energy is dissipated via vibrational motion. Photochemically triggered drug delivery systems are usually made of an ortho-nitrobenzyl photolinker, or coumarin- and pyrene-containing random copolymers with light-responsive pyrene ester bonds that irreversibly cleave upon UV irradiation (Wang et al., 2015). Mesoporous silica nanocontainers loaded with cyclodextrin were combined with photoactivation of “snap-top” stoppers over the pore openings for triggered release (Guardado-Alvarez et al., 2013). The on-command release was stimulated by UV photon activation that is suitable for use in biological systems because it enabled good tissue penetration and precise spatial control. Penetration of UV-responsive systems into the clinic is favored by the fact that light-based therapies are already being used. However, practical and regulatory issues, such as depth of tissue penetration and possible phototoxicity of the light used, are limiting the UV-triggered drug delivery system used today (Barhoumi et al., 2015). In fact, the type of light employed as well as its dosages and power have to be adjusted based on the target organs. Light-actuated drug delivery was also achieved by the reversible conformational change of molecules, such as azobenzenes, induced by irradiation with UV and visible light. These molecules contain two phenyl groups joined by N=N bond that change from *trans* to *cis* conformation once excited by UV light. The *cis* conformation relaxes with the thermodynamically stable *trans* isomer in the dark or under visible light (Dhammika Bandara and Burdette, 2012). For example, Geng et al. (2017) synthesized an azobenzene derivative, 4-cholesterocarbonyl-4′-(*N*,*N*,*N*-triethylamine butyloxyl bromide) azobenzene, and incorporated it into liposomal membranes to serve as an on–off switch of doxorubicin release. In another work, Cao et al. (2014) prepared a photoresponsive hydrogel by free radical copolymerization of xylan-type hemicellulose methacrylate with 4-[(4-acryloyloxyphenyl)azo]benzoic acid. Under UV irradiation, the *trans* conformation of azobenzene in the hydrogel convert into the *cis* conformation and resulted in the hydrophilic/hydrophobic balance variation of the hydrogel that accelerated the release of vitamin B12. Although the majority of light-triggered release platform works focused on cancer treatments, in recent years, these active materials were also designed for the delivery of growth factors for TE purposes. Photoresponsive supramolecular polysaccharide hydrogels were prepared through host–guest interactions between azobenzene and β-cyclodextrin groups conjugated to hyaluronic acid chains (Zhao et al., 2020). The hydrogel showed a decrease in the spatial network crosslink density under the application of UV light stimulus that resulted in the fast release of epidermal growth factor for wound healing. In another work, a library of polymerizable ortho-nitrobenzyl macromers with different functionalities at the benzylic position was synthesized to allow for the direct conjugation of therapeutic agent and its subsequent controlled photorelease from a hydrogel network (Griffin et al., 2013). Utilizing the photodegradable macromer incorporating an activated disulfide, the authors conjugated transforming growth factor-β1 (TGF-β1) into the hydrogel and controlled their release with light to induce chondrogenic differentiation of human mesenchymal stem cells (hMSCs).

Additional methods to trigger the release of biomolecules from biomedical devices and scaffolds aided by external activation involve the use application of electrical and/or magnetic fields as well as by acoustic and/or ultrasound stimulation (Moncion et al., 2017; Gao et al., 2019; Ahmadi et al., 2020; Lu et al., 2020; Oliva and Almquist, 2020; Thébault et al., 2020). Ultrasound-sensitive microbubbles, liposomes, and emulsions have advanced the field of ultrasound-triggered drug delivery systems as they undergo the phenomenon of cavitation and destruction followed by encapsulated drug release. This strategy was applied, among others, for improvement of angiogenesis and osteogenesis in bone defect repair with ultrasound-targeted VEGF-loaded PLGA microbubbles (Gong et al., 2019) or to trigger the release of an avascular agent, combretastatin A4 phosphate, from ultramagnetic liposomes monitored by NMR (Thébault et al., 2020). Ultrasound-triggered drug delivery emulsions were also recently loaded inside a fibrin hydrogel to spatially direct cell migration and angiogenesis in acoustically responsive fibroblast growth factor (bFGF) delivery scaffolds (Moncion et al., 2017; Lu et al., 2020). By applying spatial patterns of ultrasounds to the *in vivo* implanted scaffolds, the authors spatially controlled bFGF release to elicit a spatially directed response from the host (Lu et al., 2020). Magnetically responsive scaffolds are another important class of responsive drug delivery platforms and can be prepared by the incorporation of iron oxide nanoparticles inside a biocompatible matrix to obtain a so-called ferrogel. The basic principle of release control is that the entrapped nanoparticle moves under the effect of magnetic field and deformed the scaffolds accelerating the release of therapeutic loads (Oliva and Almquist, 2020). By using this principle, authors triggered the release of PDGF from methacrylated chondroitin sulfate-based hydrogels without inducing structure degradation. This released PDGF promoted the proliferation of human tendon-derived cells and human adipose-derived stem cells as well as the expression of tendon- and bone-related markers, respectively (Silva et al., 2018). With a similar approach, alginate ferrogels modified with heparin enabled the sustained release of TGF-β1 upon magnetic field stimulation, enhancing chondrogenic differentiation of mouse teratocarcinoma cells (Kim et al., 2016).

The works herein described highlighted some of the most used ways to control, both passively and actively, the delivery of drugs and GFs from nanocarriers and scaffolds for biomedical applications. In the next part of this review, we focus our attention on the most novel and advanced techniques that applied these drug delivery strategies to scaffolds prepared by AM, aiming to tune the spatial and temporal release of biomolecules for recreating complex biomimetic 3D systems for new tissue growth.

## Spatial and Temporal Control of Biomolecule Presentation in 3D Scaffolds Prepared by Additive Manufacturing

Advancement in TE strategies require the design of smart, functional, and high-performance scaffolds with robust and versatile manufacturing processes and capable of replicating the morphological, microstructural, and biochemical features of ECM. AM techniques revolutionized the means by which biomaterials, cells, and drugs are designed, developed, processed, and integrated and, therefore, represent the present and future of 3D drug delivery scaffold design and manufacturing.

**FIGURE 2:**
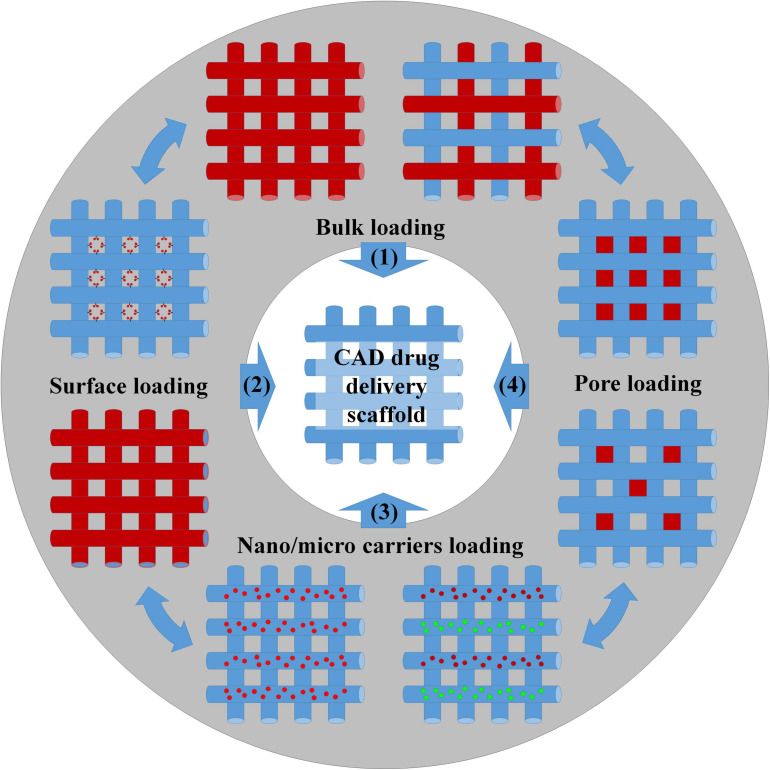
Scheme of the different methods for the preparation of drug-loading scaffolds: (1) Bulk loading involved mixing drugs and biomaterials by melt/solution blending before 3D structure fabrication or, alternatively, by wet/supercritical CO_2_ impregnation of the settled scaffold. (2) Surface bioactivation required the adsorption/grafting of the biomolecules to the scaffold surface or the incorporation of the biomolecules inside the coatings. (3) Biomolecules were loaded inside nano/microcarriers, and the carriers were further blended with the scaffold matrix before manufacturing. (4) The biomolecules were loaded into the scaffold pores using a carrier system (e.g., hydrogel).

Additive manufacturing techniques can be conveniently classified into discontinuous techniques, where layers’ fabrication and assembly involve two distinct processing steps, and continuous techniques, where these two steps are mostly automatized and take place at once (Salerno et al., 2019). Both approaches have been used in the past years to load GFs to stimulate cell growth (Bittner et al., 2018; Koons and Mikos, 2019); anti-inflammatories and immunomodulators were used to control *in vivo* body response after scaffold implantation (Zhu et al., 2020); chemotherapist molecules were delivered to kill cancer cells and stop tumor progression (Shi et al., 2020). As summarized in Figure 2, loading bioactive molecules in AM scaffolds was achieved during manufacturing or by postprocessing treatments and following four main methods.

Bulk loading (strategy n°1) requires drug/polymer blending before scaffold fabrication and represents the most common and facile strategy for obtaining bioactive polymeric scaffolds. Blends can be prepared by dissolving both compounds into organic solvents or by mixing drugs and polymers in the melt state. Melting is the preferred way to avoid the use of toxic organic solvents and when the use of high temperatures does not affect the bioactivity of the entrapped molecules. The distribution and morphology of the drug in the scaffolds depend upon the physical–chemical interaction between the drug and polymer. A favorable interaction may allow for achieving high levels of drug loading and homogeneous drug distribution. Conversely, a poor interaction resulted in phase segregation with the majority of the drug crystallized onto a scaffold surface and the difficult control over the release kinetic (Calori et al., 2020). Porous scaffolds prepared by bulk loading were developed for the purpose of regenerating complex tissues such as bone and blood vessels (Ahlfeld et al., 2019; Zhang et al., 2019; Tamjid et al., 2020). For instance, 3D printing technology was used to prepare bioresorbable vascular polylactic acid scaffolds loaded with sirolimus, to solve problems such as long-term stent restenosis (Zhang et al., 2019). Sirolimus is a natural macrocyclic lactone, which inhibits smooth muscle cell proliferation and migration to reduce neointima formation and stent stenosis (Jelonek et al., 2018). Mixing the drug with scaffold preparation material in solution ensured reducing burst release and, consequently, avoided possible acute cytotoxicity to the surrounding tissues. The implant enabled a sustained release up to 16 months *in vivo* providing the required therapeutic treatment. Bulk loading combined with a 3D printing process was also applied to fabricate biphasic scaffolds for the spatial–temporal controlled release of VEGF toward bone regeneration (Ahlfeld et al., 2019). The scaffold was obtained by extrusion-based 3D multichannel plotting of a calcium phosphate cement paste and a VEGF-loaded alginate/gellan gum (AlgGG) hydrogel paste. The outer geometry of the biphasic scaffold was designed as a cylinder with a 5-mm diameter base to fit in the femur diaphysis of rats and make tight contact with the osteosynthesis plate. A triangular pore structure with 60° strand orientation was used in the inner architecture design, while the scaffolds had a gradient of VEGF-loaded AlgGG strands, increasing from the outer to the inner scaffold regions. The scaffold revealed good handling and fitting properties as well as bone tissue ingrowth and vascularization in response to locally released VEGF (Ahlfeld et al., 2019). As previously discussed, drug impregnation postprocessing can be classified depending on the drug impregnation medium into wet and supercritical impregnation strategies. The first strategy was applied, for instance, to load VEGF onto laponite–alginate–methylcellulose bone hydrogel scaffolds encapsulating human bone marrow stromal cells (Cidonio et al., 2020). In the treatment of acute and chronic skin loss conditions, such as venous ulcer and diabetes, the development of skin grafts may allow for overcoming possible donor site morbidity and immune-rejection problems often occurring when using autografts and allografts. A 3D-printed gelatin patch coated with sulfonated silk fibroin derivative was developed to serve as a “porous magnet” to sequester and concentrate basic fibroblast growth factor (FGF-2) and to promote the formation of granulation tissue and enhance the repair of full-thickness skin defects (Xiong et al., 2017). Incorporation of FGF-2 within the scaffold was obtain by soaking the scaffold in FGF-2 solution, while its release enhanced cell proliferation rate, tissue morphology, collagen fibril assembly, and blood vessel formation, and demonstrated great potential for major cutaneous defects, such as repairing large-area skin damage and chronic skin wounds due to lower granulation (Xiong et al., 2017). However, wet absorption is often unsuitable for loading bioactive molecules into scaffolds made of thermoplastic polymers due to slow solution diffusion into the bulk. By using scCO_2_, Ngo et al. (2020) fabricated flurbiprofen-loaded acrylate-based 3D-printed systems and modulated the amount of loaded drug in the range of 12.72–24.08% by varying the operating temperature and pressure. Concomitantly, 3D-printed scaffolds processed with scCO_2_ enabled the tuning of surface roughness features and macro/microporous porosities for specific application needs (Zhou et al., 2016; Ngo et al., 2020). Surface loading (strategy n°2) of bioactive molecules requires scaffold postprocessing treatments similar to the wet and vapor treatments described previously. In fact, biomolecule loading depended on their physical or chemical adsorption onto the scaffold pore surface, while biomolecule delivery depended on the interaction between scaffold material and drug, the specific surface, and the diffusion of the release medium into the scaffold core. An example of this approach was the work by Saska et al. (2018) that investigated the postprinting functionalization with osteogenic growth peptide (OGP) and its C-terminal sequence OGP(10–14) of poly(3-hydroxybutyrate) scaffolds. OGP peptide loading was carried out by immersing the scaffolds into a peptide solution for 72 h at 10°C followed by air drying at 37°C. Similarly, Tamjid et al. (2020) loaded tetracycline hydrochloride into PCL composite scaffolds to enhance its antibacterial properties, while Gbureck et al. (2007) bioactivated bioceramic bone implants with spatially localized angiogenic factors. If compared with bulk loading, surface bioactivation has some important advantages. In fact, the bioactivation of porous scaffolds by postprocessing enabled overcoming problems related to possible biomolecule deactivation that may conversely occur during drug–polymer premixing and scaffold manufacturing. Besides, by carrying out scaffold manufacturing and drug loading into two independent steps, it was possible to expand material choice and scaffold formulation possibilities and incorporate hydrophilic compounds into hydrophobic scaffolds. For example, hollow poly(lactic acid) (PLA) scaffolds prepared by fused deposition modeling were coated by solution-casting mixtures of differing molecular weights of PCL and poly(ethylene glycol) (PEG) (Stewart et al., 2020). These implants demonstrated *in vitro* release rates for hydrophilic model compounds (methylene blue and ibuprofen sodium) that were modulated in a facile way by changing the formulation of the polymeric coating. However, if compared with bulk loading, scaffolds prepared by surface bioactivation usually evidenced lower drug loading and limited control of drug release kinetic and spatial distribution. Overcoming the limitations required the increase of the specific surface of the scaffolds, for instance, by creating bimodal macro-microporosity (Visscher et al., 2018; Liu et al., 2020) or by grafting the biomolecules to the polymeric chains. Nevertheless, the difficult control of the contact points between solution/vapor carrying biomolecules and scaffold surfaces hindered the fabrication of scaffolds having spatial gradients of bioactive factors.

In order to achieve spatiotemporal delivery, recent advances in AM of scaffolds have paved the way for incorporating micro or nanoparticles loaded with biomolecules inside scaffolding material (strategy n°3) (Fahimipour et al., 2017; Zhu et al., 2018; Guzzi et al., 2019). Indeed, these carriers not only protect the encapsulated molecules against solvents and temperature during processing but also release the molecule in a sustainable manner. Furthermore, their localization inside the scaffold was controlled during manufacturing, finally resulting in a spatial and temporal controlled release. In order to optimize VEGF release timing at the preferred location within 3D bioprinted scaffolds, Poldervaart et al. (2014) fabricated Matrigel scaffolds containing human endothelial progenitor cells (EPCs) and VEGF-loaded gelatin microparticles. These scaffolds allowed a sustained VEGF release and enhanced vessel formation after implantation in subcutaneous pockets in nude mice. The use of microcarriers was also implemented to achieve sequential release function of chemokine stromal cell-derived factor-1 (SDF-1) and Y27632 factors from polyurethane scaffolds for cartilage TE (Wen et al., 2019). The fast release of SDF-1 attracted MSCs from the surrounding tissues, while the later release of Y27632 factor stimulated MSCs differentiation into chondrocytes. Microprecise spatiotemporal delivery scaffolds were achieved by the proper choice of microspheres and scaffold strut materials. For instance, the formation of multitissue interfaces from bone marrow-derived mesenchymal stem/progenitor cells was achieved by controlling the localization of PLGA microspheres loaded with connective tissue growth factor (CTGF) and transforming growth factor β3 (TGF-β3) inside PCL scaffolds (Tarafder et al., 2016). Given the substantial difference in the melting points between PLGA and PCL and their low heat conductivity, the microsphere structure was not altered during the process, protecting biomolecules from thermal degradation. This microprecise spatial control of multiple GFs was achieved by interchanging dispensing cartridges during a single printing process, and the as-prepared scaffolds significantly prevented arthritic changes on temporomandibular joint condyles (Tarafder et al., 2016).

The last strategy (n°4) to bioactivate porous scaffold is to fill the pores with a carrier material loaded with the biomolecules. Although this approach reduces scaffold porosity for cells and tissue ingrowth, it was suitable to control drug delivery behavior and to impart additional features to porous scaffolds made of thermoplastic materials or bioactive ceramics. For example, beta-tricalcium phosphate scaffolds with CAD designed structure were filled with a collagen–heparin thermogel encapsulating both BMP-2 and MSCs to enhance bone regeneration (Fahimipour et al., 2019). The heparin-functionalized collagen gel retained the bioactivity of growth factors and supported MSC viability and differentiation. Concomitantly, the ceramic fibers ensured the adequate mechanical support and the correct integration with surrounding bone tissue. Tuning hydrogel properties allowed for the development of composite scaffolds providing drug release and on-demand photothermal conversion functions (Jiang et al., 2020). This approach was also used to obtain miniaturized modular LEGO-like cage scaffolds loaded with biologic cargo of different compositions and assembled into highly complex structures to pattern therapeutics within the material in 3D (Hipfinger et al., 2020). It is worth noting that all of the approaches described in Figure 2 can also be combined to others aiming to increase scaffold design complexity. For example, a collagen type 1 solution containing PLGA microspheres loaded with VEGF, BMP-2, or FGF-2 was incorporated into the pores of polycaprolactone fumarate scaffolds and crosslinked under UV light to stimulate vascular ingrowth and tissue regeneration (Wagner et al., 2018). Growth factor-loaded microspheres were also deposited on the surface of melt electrowriting scaffold pores by an inkjet spray drying technique to prepare three layers of scaffolds for repairing cartilage injury (Han et al., 2020). The scaffold consisted of a surface layer loaded with BMP-7 and TGF-β1, a middle layer loaded with IGF-1 and TGF-β1, and a deep layer loaded with hydroxyapatite (HA) and TGF-β1. This design stimulated the adhesion, proliferation, and differentiation of MSCs recruited from the bone marrow and blood, while contributing to the regional heterogeneity of chondrocytes and secreted proteins to promote functional cartilage regeneration. Novel scaffolds performing multidrug spatiotemporal release were also engineered by filling the pores of bioprinted scaffolds with electrospun nanofibers loaded with biomolecules (Liu et al., 2016). These scaffolds provided a biomimetic nanofibrous pore morphology to support cell growth and enhance cell retention, while ensuring the controlled delivery of growth factors and other drugs for tissue regeneration.

## Bioprinting of Bioactive Hybrid Scaffolds for Musculoskeletal Tissues

Musculoskeletal tissue damage and degeneration as a consequence of traumas and/or diseases are common and debilitating events that cannot be often healed by one’s own body tissue regeneration capability due to extensive inflammation and the high degree of damage (Loebel and Burdick, 2018). The high prevalence of musculoskeletal tissue injuries has directed significant investments in the development of TE therapies to enhance healing of damaged musculoskeletal tissues, such as bone, cartilage, and osteochondral tissues. However, the biologically and architecturally complex composition and structure of these tissues are challenging goals for TE. For example, bone is a connective tissue characterized by a multitude of mechanical, chemical, and hematological functions. Furthermore, bone is subjected to continuous remodeling based on time- and spatial-dependent physiological changes (Hutmacher et al., 2007). From a material point of view, bone is a natural composite consisting mainly of a collagen organic phase and a hydroxyapatite inorganic phase. The interaction and balance between these two phases are responsible for the biomechanical properties of bone tissue, characterized by elastic compression moduli in the 18- to 20-GPa range (Bayractar et al., 2004). Bone tissue intraosseous vasculature is highly organized and ensure essential nutrients to closed osteocytes and allow the removal of cellular metabolic wastes (Santos and Reis, 2020). Articular cartilage is a highly organized tissue that provides a low-friction and wear-resistant bearing surface and exhibits regional organization, e.g., structure, cells, and ECM biochemical composition, to match biomechanical requirements (Steele et al., 2014). Indeed, the superficial zone exhibits a collagenous fibrous structure aligned to the surface and rich in chondrocytes to ensure high tensile strength upon wearing and a deeper region richer in proteoglycan concentration while reducing cell concentration to guarantee cartilage compression resistance by producing a high osmotic pressure within the tissue (Steele et al., 2014).

To fabricate biomimetic tissues, with zone-specific heterogeneity like musculoskeletal tissues, multimaterial and multicell-type bioprinting with micrometric scale control of localization is demanding. Bioprinting allows the fabrication of patient-specific, implantable 3D constructs by using in a simultaneous and controlled way cartridge loaded with different matters: biomaterials in form of pastes, polymeric composite melt or solution; free drug or drug-encapsulated carriers; and cells of different origins in suspension or encapsulated within hydrogels. Bioprinting techniques can be broadly classified into three main categories (Murphy et al., 2017): (i) laser-assisted, (ii) inkjet-based, and (iii) extrusion-based printing. To date, extrusion-based 3D bioprinting is the most successful biofabrication process as cells, hydrogels, and other materials are deposited onto a substrate by using one or multiple pressurized syringes. The pressure system consists of either a mechanical piston or a pneumatic pressure source (mostly compressed air) that is computer-controlled. Besides, through bioprinting, it was possible to design and fabricate evenly complex hybrid scaffolding systems to mimic biological tissue hierarchical architecture and composition and suitable to enhance tissue regeneration potential.

Table 1 highlights some of the most recently published work on the engineering of musculoskeletal tissue scaffolds with spatial and temporal controlled release capability by means of bioprinting technique. In a recent study, a multiple-tool biofabrication technique was used to deliver VEGF and BMP-2 with distinct spatiotemporal release profiles from porous composite scaffold made of PCL and alginate to enhance the regeneration of critically sized bone defects (Freeman et al., 2020). The fabrication process started by printing a PCL structural scaffold (4-mm diameter and 5-mm height) having both lateral and horizontal porosity, and a fiber spacing of 1.2 mm. The scaffold was subsequently loaded with two different alginate-based nanocomposite bioinks. The vascular bioink, consisting of 3.5% w/v RGD-alginate, 1.75% w/v methylcellulose, 3.5% w/v nHA, and 500 ng/ml VEGF in alpha minimum essential medium (αMEM), 10% fetal bovine serum (FBS), penicillin (5% v/v), and streptomycin (5% v/v), was loaded in the scaffold center to stimulate blood vessel ingrowth. The osteoinductive bioink, consisting of 3.5% w/v RGD-alginate, 1.75% w/v methylcellulose, 0.5% w/v laponite, and 10 μg/ml of BMP-2 solubilized in the previously described medium, was loaded in the periphery to promote bone growth and implant integration with surrounding tissue. A proof-of-concept study in nude mice validated the benefit of this precise localization of growth factors in both time and space on angiogenesis and new tissue formation (Freeman et al., 2020). In fact, the composite scaffold demonstrated accelerated bone defect healing with higher levels of vessel invasion and less heterotopic bone formation if compared with implants homogeneously loaded with the same total amount of growth factors. Similar hydrogel-PCL composite scaffold strategies were proposed by the group of Sun et al. (2020a,b, 2021) to generate living anisotropic cartilaginous tissues (Figure 2). In the case of meniscus, PCL was molten to fabricate the physically supporting structure for the scaffold, choosing needle diameter, layer thickness, and fiber spacing of 200, 200, and 350 μm, respectively. Furthermore, inspired by the heterogeneity of native meniscus structure, a composite hydrogel was made mixing gelatin (45 mg/ml), fibrinogen (30 mg/ml), hyaluronic acid (30 mg/ml), and glycerol (10% v/v) and loaded with MSCs and PLGA microparticles carrying connective tissue growth factor (CTGF) and TGF-β3. These growth factors induced differentiation of MSCs into fibrochondrocytes and were located in different porous regions of the scaffold. In particular, to chemically simulate the anisotropic phenotypes in native meniscus, microcarriers carrying CTGF were positioned in the outer one-third region, while those carrying TGF-β3 were used for the inner two-thirds regions of the meniscus construct. *In vivo* implantation into sheep showed that the ECM composition of the 3D-bioprinted constructs shared many characteristics of native meniscus, including the heterogeneous zonal expression of types I, II collagen and therewith the conferred anisotropic zonal function properties (Sun et al., 2020a). Dual-factor releasing and gradient-structured bioprinted constructs were also used for anisotropic cartilage regeneration (Sun et al., 2020b). As native articular cartilage transitions from the superficial zone to the deep zone, gradient anisotropic cartilage scaffold was constructed by one-step 3D bioprinting gradient polymeric scaffolding structure. The gradient PCL fiber spacing ranged gradually from 150 μm of the superficial zone of the cartilage, providing higher mechanical properties and smaller pores for chondrocyte differentiation, up to 750-μm pores in the construct core to enhance diffusion of nutrients and vessel ingrowth. Furthermore, as in the case of meniscus construct, dual protein-releasing composite hydrogels encapsulating MSCs and PLGA microspheres loaded with either TGF-β3 and BMP-4 were bioprinted into the pores between PCL fibers (Sun et al., 2020b). Specifically, the BMP-4 hydrogel was located in the deepest layer with a 750-μm PCL fiber spacing, while the TGF-β3 hydrogel was used for the other three layers of the cartilage construct.

**TABLE 1:** Examples about the use of the bioprinting technique to fabricate complex drug delivery scaffolding systems for the regeneration of musculoskeletal tissues.

Tissue	Bioactive scaffold			Outcome	References
	Design features		Composition		
Bone	Structural support	Cylindrical construct (*d* = 4 mm and *h* = 5 mm) with 1.2-mm fiber spacing	PCL	High vessel invasion and accelerated large bone defect healing with little heterotopic bone formation	Freeman et al. (2020)
	Delivery system	Osteoinductive composite hydrogel printed in the pores of the periphery	RGD-modified alginate, methylcellulose, and laponite		
		Vascular composite hydrogel printed in the pores of the center	RGD-modified alginate, methylcellulose and hydroxyapatite nanoparticles		
	Biological component	No cells	/		
Cartilage	Structural support	Four-layer graded cubic scaffold (*l* = 4 mm) with fiber spacing varying from 150-μm wide from the superficial zone to 750-μm wide in the deep zone of the cartilage construct	PCL	Whole-layer integrity, lubrication of superficial layers, nutrient supply in deep layers, and cartilage tissue maturation suitable for translation to patients	Sun et al. (2020a)
	Delivery system	Chondrogenic microsphere-laden hydrogel printed in the pores of the first three layers	Composite hydrogel made of gelatin, fibrinogen, hyaluronic acid, and glycerol and incorporating polylactic-co-glycolic acid (PLGA) microspheres encapsulating transforming growth factor-β1 (TGF-β3)		
		Osteoinductive microsphere-laden hydrogel printed in the pores of the deepest layer with a 750-μm PCL fiber spacing	Composite hydrogel made of gelatin, fibrinogen, hyaluronic acid, and glycerol and incorporating PLGA microspheres encapsulating bone morphogenetic protein-2 (BMP-4)		
	Biological component	Cell-laden osteoinductive and chondrogenic bioinks	Bone marrow-derived mesenchymal stem cells (MSCs)		
Meniscus	Structural support	Anatomically shaped meniscus structure with fiber size of 200 μm and fiber spacing of 350 μm	PCL	Goat anisotropic meniscus construct having the heterogeneous zonal expression of types I, II collagen and ready for implantation	Sun et al. (2020b)
	Delivery system	Chondrogenic microsphere-laden hydrogel printed in the pores of the inner 2/3 region of the meniscus construct	Composite hydrogel made of gelatin, fibrinogen, hyaluronic acid, and glycerol and incorporating PLGA microspheres encapsulating TGF-β3		
		Chondrogenic microsphere-laden hydrogel printed in the pores of the outer 1/3 region of the meniscus construct	Composite hydrogel made of gelatin, fibrinogen, hyaluronic acid, and glycerol and incorporating PLGA microspheres encapsulating connective tissue growth factor (CTGF)		
	Biological component	Cell-laden chondrogenic bioinks	Bone marrow-derived MSCs		
Intervertebral disk (IVD)	Structural support	Anatomically shaped IVD scaffold consisting of five parts: (1) the upper cartilage endplate; (2) the lower cartilage endplate; (3) the nucleus pulposus; (4) the annulus fibrous, and (5) the annulus fibrous support	PCL	The reconstructed IVD scaffold exhibited a zone-specific matrix phenotype with type II collagen and glycosaminoglycan in the core zone, and type I collagen in the surrounding zone	Sun et al. (2021)
	Delivery system	Nucleus pulposus bioink printed in the pores of the nucleus pulposus	Composite hydrogel made of gelatin, sodium alginate, and hyaluronic acid and loaded with polydopamine nanoparticles encapsulating TGF-β3		
		Annulus fibrous bioink printed in the pores of the annulus fibrous	Composite hydrogel made of gelatin, sodium alginate, and hyaluronic acid and loaded with polydopamine nanoparticles encapsulating CTGF		
	Biological component	Cell-laden nucleus pulpous and fibrous annulus bioinks	Bone marrow-derived MSCs		
Osteochondral	Structural support	Cylindrical construct (*d* = 6 mm; *h* = 5 mm) with 160-μm fiber diameter and 250-μm fiber spacing	PCL	Gene-activated bioprinted construct supported the vascularization and mineralization in the osseous region, while sGAG and type II collagen-rich cell clusters formation in the cartilage region	Gonzalez-Fernandez et al. (2019)
	Delivery system	Osteogenic bioink casted in the bottom layer of 4 mm	Alginate-methyl cellulose composite hydrogel containing nanohydroxyapatite particles-pBMP-2 complexes		
		Chondrogenic bioink casted in the top layer of 2 mm	Alginate-methyl cellulose hydrogel containing RALA–pTGF-β3–pBMP2–pSOX9 complexes		
	Biological component	Cell-laden osteogenic and chondrogenic bioinks	Bone marrow-derived MSCs		

The versatility of the bioprinting strategy combining a structural support made of a thermoplastic polymer (PCL) and drug delivery composite hydrogels incorporating GF-loaded carriers was demonstrated by the same group to engineer also an anatomically correct intervertebral disk (IVD) scaffold (Sun et al., 2021). Connective tissue growth factor (CTGF) and TGF-β3 were loaded into polydopamine nanoparticles mixed with MSCs for regenerating and simulating the structure and function of the nucleus pulposus and annular fibrous. A 3D virtual model of the IVD scaffold was designed into five parts: (1) the upper cartilage endplate, (2) the lower cartilage endplate, (3) the nucleus pulposus, (4) the annulus fibrous, and (5) the annulus fibrous support. The CTGF/MSCs ink and TGF-β3/MSCs ink were loaded into the annulus fibrous and nucleus pulposus parts, respectively. *In vivo* experiments confirmed that the reconstructed IVD scaffold exhibited a zone-specific matrix phenotype, as the TGF-β3 promoted the biosynthesis of glycosaminoglycan and collagen II in the nucleus pulposus, while the CTGF stimulated the biosynthesis of glycosaminoglycan and collagen I in the annulus fibrous (Sun et al., 2021).

Engineering cells to synthesize and deliver *in situ* growth factors through gene delivery represents an alternative approach to direct stem cell fate within the tissue construct. Non-viral gene-activated bioprinted scaffolds providing a temporal and spatial control of plasmid gene delivery to stem cells were developed to engineer an osteochondral implantable cell-laden construct consisting of a cartilaginous matrix overlaying a vascularized bone tissue (Gonzalez-Fernandez et al., 2019). The newly developed bioink was obtained by blending sacrificial and stable hydrogels, providing an active platform to temporally modulate transfection of host or transplanted cells *in vivo* by increasing scaffold porosity over time with transient or sustained rates (Table 1) (Gonzalez-Fernandez et al., 2019). In particular, the bioinks containing stem cells and plasmids encoding for either osteogenic (BMP-2) or chondrogenic (combination of TGF-β3, BMP-2, and SOX9) genes were printed inside specific porous zones of 3D-printed PCL scaffold, and the composite constructs guided the formation of a vascularized, bony tissue overlaid by a layer of stable cartilage (Gonzalez-Fernandez et al., 2019).

Although all of the previous reported works clearly demonstrated the advancement of bioprinting in musculoskeletal tissue reconstruction, researchers working on tissue bioprinting have to face up to two major limitations in the future (Sigaux et al., 2019). First, there are so many options in bioink composition and patient-specific tissue properties that defining a unique strategy for each tissue is complex. Vascularization of the printed tissues is the other main challenge as cells and tissues cannot survive without adequate blood circulation, and integrating a full vascular network (from large vessels to capillaries) into the printed tissues is still a challenge. Once the vascularization of the *in silico* designed tissues is overcome, the translation of bioprinted tissues to personalized medical treatments and reconstructed surgery will be possible by a two-step management for patients (Sigaux et al., 2019). In a first 1-day appointment, the patient is subjected to specific biopsies to obtain autologous cell sources for tissue printing and maturation *in vitro*. Then, a second step surgery is performed to implant the *in vitro* grew tissue.

## Recent Applications of Computer-Aided Design Drug Delivery Platforms for Cancer Treatment

Cancer is one of the major causes of morbidity and mortality worldwide, leading to significantly increased healthcare costs and the great need to better understand cancer to improve therapy (Shrike Zhang et al., 2016). The biochemical (e.g., growth factors and cytokines) and biophysical cues (e.g., ECM mechanics) of tumor microenvironment are highly complex and dynamic and play a significant role in tumor growth and metastasis development (Shrike Zhang et al., 2016). The application of TE scaffold-based strategies toward cancer genesis and treatment are therefore highly desirable as they could help in understanding *in vitro* how cancer cells and the ECM become implicated in tumor growth and migration, and they can be used in the clinic to stimulate tissue repair after tumor resection and reduce tumor cell migration risks (Katt et al., 2016; Mao et al., 2020; Oztan et al., 2020; Shafiee, 2020). Scaffolds for the treatment of human tissue defects after tumor resection required loading and release of chemotherapy molecules for residual tumor cell suppression after surgery. In fact, if compared with high-dose intravenous chemotherapy, drug-loaded implants have the advantages of single-drug administration, minimal systemic toxicity, and increased delivery efficacy. Furthermore, when fabricated starting from 3D reconstruction images of critical size tissue defect, these patient-specific implants served as space holders to prevent undesired tissue invasion from the immediate vicinity into the affected site and simultaneously provided a temporary biomechanical support for the growing tissue and sustain *in vivo* loads. To address these issues for postsurgical bone tumor management, a multifunctional bone graft substitute was designed by incorporating the soy isoflavones genistein, daidzein, and glycitein in a 5:4:1 ratio, onto a 3D-printed tricalcium phosphate (TCP) porous scaffold (Sarkar and Bose, 2020). The TCP scaffold was designed as having an interconnected porosity and biodegradation rate to control isoflavone release kinetics. Most importantly, genistein delivery was designed to reduce osteosarcoma cell viability and proliferation, while daidzein and glycitein promoted osteoblast attachment, viability, and proliferation *in vivo* into a critical-sized bicortical defect in the lateral epicondyle. The efficacy of AM scaffolds for local release of chemotherapist for osteosarcoma treatment was also demonstrated *in vitro* by using composite scaffolds made of silica nanoparticles and PCL incorporating ruthenium-loaded PEGylated liposomes (Ye et al., 2019). The authors found that the scaffolds had a relatively slow sustained chemotherapist release and a good antitumor efficacy over a relatively long period. The use of porous scaffolds as local drug reservoirs to prevent cancer recurrence and stimulate new tissue regeneration was also suitable for soft tissues applications, such as breast cancer therapy (Dang et al., 2020; Yang et al., 2020). AM and salt-leaching techniques were combined to produce bimodal porous PCL scaffolds that were subsequently loaded with doxorubicin by the wet dipping method. The scaffolds displayed a chemotherapeutic effect against breast cancer cells and, if compared with systemic administration, reduced local cancer recurrence and showed lower cardio-cytotoxicity effect (Dang et al., 2020). Similarly, PLGA scaffolds fabricated by 3D printing and loaded with anticancer molecules significantly reduced the required drug dosages and ensured curative drug levels near tumor sites for prolonged periods, while drug exposure to normal tissues was minimized (Yang et al., 2020).

The utilization of CAD-based processes for *in vitro* creation of tumor models is widespread as it enabled testing drug efficacy and studying tumor growth and progression mechanisms. For example, modeling tumor microenvironments through bioprinting had the potential to overcome limitations related to cancer study on 2D systems and/or cell spheroids thanks to its freeform nature, adaptability, customizability, scalability, and diversity (Salerno and Netti, 2014; Oztan et al., 2020). Existing bioprinting methods used in cancer research involved extrusion, stereolithography, and inkjet printing techniques and have significantly improved accuracy and composition of tumor environment design and, therefore, drug testing reliability and scale-up to humans. Bioprinted tumor models were fabricated to mimic *in vitro* the physical and cellular properties of cancer of tissues like the breast, brain, bone, and lung (Kang et al., 2020; Radhakrishnan et al., 2020). For example, a series of 3D bone matrices of variable geometry were printed using stereolithography and used to study breast cancer cell growth (Zhu et al., 2016). It was found that matrix geometry and composition, together with the coculture of breast cancer cells and MSCs, influenced cell proliferation and enhanced cell migration capability (Zhu et al., 2016). Although 3D cancer models contribute to the recapitulation of important features of cancers and may represent suitable alternatives of the animal-based models, their standardization is still far to be possible (Shafiee, 2020). In fact, as discussed in the previous section, both cancer tissue heterogeneity and experimental processing conditions make it difficult to define standardized models, and the analysis of the mechanisms involved in cancer development are often incomplete. Concomitantly, scientific literature about drug-loaded scaffolds for a tumor model is limited, and future advances in this field depend on the efforts that will be done to integrate knowledge from cancer cell biology and drug delivery scaffold biofabrication to engineer patient-specific tumor tissue models for immediate translation to clinical applications.

## Conclusion and Future Perspectives

In this review, we described the state-of-the-art of drug delivery scaffolds prepared by AM processes for TE and cancer precision medicine. Common strategies for porous scaffolds and hydrogel bioactivation were overviewed to elucidate the importance of material selection together with 3D structure design on drug loading and delivery efficacy. To date, there is a plethora of drugs and biomolecules, such as GFs and anti-inflammatories, that can be incorporated inside porous scaffolds to guide cellular processes involved in new tissue regeneration. Among them, VEGF is an excellent biomolecule to enhance the vasculogenic potential of the scaffold, especially when combined with PDGF and BMP. However, it was demonstrated that the efficacy of these molecules depend on their bioactivity, their presentation to cell-surface receptors, and their spatial and temporal controlled release. In view of these important aspects, great attention must be paid to the way these biomolecules are incorporated into the scaffolds, in order to avoid possible deactivation as well as to maximize biomolecule loading and achieve a sustained release over the entire time required for the biological stimulation process. The use of drug-loaded micro and nanocarriers have opened new possibilities for scaffold design as they allow for enhanced control over scaffold release features, together with the possibility of protecting the bioactive molecules during scaffold manufacturing, especially when high temperature and/or aggressive solvents are used. Besides, these carriers allow for loading multiple biomolecules inside porous scaffolds and test the efficacy of synergic biomolecule delivery on scaffold biocompatibility and integration into the host body. The development of advanced biomaterials whose properties can be adjusted by variation of biophysical and biochemical conditions, such as changes in temperature or magnetic field, has also opened new routes to enhance therapeutic efficacy of biomolecule-releasing scaffolds.

It is universally recognized that, among the different AM scaffold fabrication processes, bioprinting represents, nowadays, the most powerful technique addressing patient-specific demand for tissue repair mediated by drug delivery implantable scaffolds. In fact, this technique enabled the manipulation of almost any kind of material, spanning from thermoplastic polymers to hydrogels and ceramic pastes, cells, and biomolecules such as GFs, and create evenly complex 3D structures mimicking the composition, architecture, and functionalities of the native ECM. The importance of the bioprinting technique in biomedicine was demonstrated by recent works applying this technique to design and manufacture ECM-mimicking scaffolds for the regeneration of complex musculoskeletal tissues. Bioprinting is also the first choice in cancer precision medicine when tissue regeneration must be achieved after tumor resection or to study chemotherapist efficacy against tumors in 3D *in vitro* models.

Despite these advancements, the translation of bioactive delivering scaffolds from bench to bedside is still a challenging goal, and further efforts are necessary to design and fabricate scaffolds providing ECM guidance functions that are suitable to successfully regenerate tissue analogs for clinical demand. It is, however, worth noting that technological advancement in the fields of materials science, cellular therapy, and drug discovery can boost AM processes advancement toward the next generation of drug delivery scaffold development. For instance, the integration of nanotechnology (e.g., soft lithography), micro/nanofluid, and bioprinting is a promising approach to enhance the control of scaffold processing/structure/delivery (Davoodi et al., 2020; Richard et al., 2020). Indeed, the formation of multiple emulsions within microfluidic devices may enable the fabrication of microparticles with multiple cores and drug/cell loading and delivery capability (Omidi et al., 2020; Tomeh and Zhao, 2020; Moreira et al., 2021). Similarly, lithography-based processes, such as those using UV-photopolymerization or patterned polydimethyl siloxane molds, offer the possibility for precise structuring drug and cell delivery microcontainers (Salerno et al., 2019; Mirza and Saha, 2020; Saraswat et al., 2020). These microcontainers may be charged with multiple drugs and biomolecules in powder form or by using scCO_2_ processing to protect the bioactive ingredient against degradation and deactivation and achieve full loading efficiency (Marizza et al., 2014; Abid et al., 2017). Furthermore, the combination of monodisperse porosity and enhanced diffusion in an even nanometric volume together with the possibility of integrating stimuli-responsive components for triggered drug delivery may allow the precise tuning of biomolecule release profiles (Randall et al., 2007; McHugh et al., 2017). All of these novel-designed carriers can be further incorporated into the bioprinted scaffold structure, inside the filament otherwise located into the pores aided by micromanipulation systems (Mekhileri et al., 2018; Hacohen et al., 2020). The as-engineered scaffolds can achieve, in principle, the nanometric scale control of biomolecule loading, and their programmed/triggered release following cell and tissue demands can finally have a tremendous impact on the production of customized clinical-grade functional tissues.

## Author Contributions

AS conceptualized and visualized the study, and wrote the manuscript. PN conceptualized the study and wrote the manuscript. Both authors contributed to the article and approved the submitted version.

## Conflict of Interest

The authors declare that the research was conducted in the absence of any commercial or financial relationships that could be construed as a potential conflict of interest.

## References

[B1] Abid Z. Gundlach C. Durucan O. von Halling Laier C. Hagner Nielsen L. Boisen A. (2017). Powder embossing method for selective loading of polymeric microcontainers with drug formulation. *Microelectron. Eng.* 171 20–24. 10.1016/j.mee.2017.01.018

[B2] Ahlfeld T. Schuster F. P. Föster Y. Quade M. Akkineni A. R. Rentsch C. (2019). 3D plotted biphasic bone scaffolds for growth factor delivery: biological characterization in vitro and in vivo. *Adv. Healthc. Mater.* 8:1801512. 10.1002/adhm.201801512 30838778

[B3] Ahmadi A. Hosseini-Nami S. Abed Z. Beik J. Aranda-Lara L. Samadian H. (2020). Recent advances in ultrasound-triggered drug delivery through lipid-based nanomaterials. *Drug Discov. Today* 25 2182–2342. 10.1016/j.drudis.2020.09.026 33010479

[B4] Barati D. Ramin Pajoum Shariati S. Moeinzadeh S. Melero-Martin J. M. Khademhosseini A. Jabbari E. (2016). Spatiotemporal release of BMP-2 and VEGF enhances osteogenic and vasculogenic differentiation of human mesenchymal stem cells and endothelial colony-forming cells co-encapsulated in a patterned hydrogel. *J. Control. Release* 223 126–136. 10.1016/j.jconrel.2015.12.031 26721447 PMC4724464

[B5] Barhoumi A. Liu Q. Kohane D. S. (2015). Ultraviolet light-mediated drug delivery: principles, applications, and challenges. *J. Controlled Release* 219 31–42. 10.1016/j.jconrel.2015.07.018 26208426

[B6] Bayractar H. H. Morgan E. F. Niebur G. L. Morris G. E. Wong E. K. Keaveny T. M. (2004). Comparison of the elastic and yield properties of human femoral trabecular and cortical bone tissue. *J. Biomech.* 37 27–35. 10.1016/S0021-9290(03)00257-4 14672565

[B7] Biondi M. Ungaro F. Quaglia F. Netti P. A. (2008). Controlled drug delivery in tissue engineering. *Adv. Drug Deliv. Rev.* 60 229–242. 10.1016/j.addr.2007.08.038 18031864

[B8] Bittner S. M. Guo J. L. Mikos A. G. (2018). Spatiotemporal control of growth factors in three-dimensional printed scaffolds. *Bioprinting* 12:e00032. 10.1016/j.bprint.2018.e00032 31106279 PMC6519969

[B9] Borselli C. Storrie H. Benesch-Leec F. Shvartsman D. Cezar C. Lichtman J. W. (2010a). Functional muscle regeneration with combined delivery of angiogenesis and myogenesis factors. *Proc. Natl. Acad. Sci. U.S.A.* 107 3287–3292. 10.1073/pnas.0903875106 19966309 PMC2840452

[B10] Borselli C. Ungaro F. Oliviero O. d’Angelo I. Quaglia F. La Rotonda M. I. (2010b). Bioactivation of collagen matrices through sustained VEGF release from PLGA microspheres. *J. Biomed. Mater. Res.* 92A:94. 10.1002/jbm.a.32332 19165799

[B11] Bruggeman K. F. Wang Y. Maclean F. L. Parish C. L. Williams R. J. Nisbed D. R. (2017). Temporally controlled growth factor delivery from a self-assembling peptide hydrogel and electrospun nanofibre composite scaffold. *Nanoscale* 9 13661–13669. 10.1039/C7NR05004F 28876347

[B12] Caballero-Aguilar L. M. Moraes Silva S. Moulton S. E. (2020). “Three-dimensional printed drugdelivery systems,” in *Engineering Drug Delivery Systems*, eds Seyfoddin A. Dezfooli S. M. Greene C. A. (Amsterdam: Woodhead Publishing), 147–162. 10.1016/B978-0-08-102548-2.00006-8

[B13] Calori I. R. Braga G. da Costa Carvalho de Jesus P. Bi H. Tedesco A. C. (2020). Polymer scaffolds as drug delivery systems. *Eur. Polym. J.* 129:109621. 10.1016/j.eurpolymj.2020.109621

[B14] Campbell K. T. Stilhano R. S. Silva E. A. (2018). Enzymatically degradable alginate hydrogel systems to deliver endothelial progenitor cells for potential revasculature applications. *Biomaterials* 179 109–121. 10.1016/j.biomaterials.2018.06.038 29980073 PMC6746553

[B15] Cantón I. Mckean R. Charnley M. Blackwood K. A. Fiorica C. Ryan A. J. (2010). Development of an ibuprofen-releasing biodegradable PLA/PGA Electrospun scaffold for tissue regeneration. *Biotechnol. Bioeng.* 105 396–408. 10.1002/bit.22530 19731254

[B16] Cao L. Mooney D. J. (2007). Spatiotemporal control over growth factor signaling for therapeutic neovascularization. *Adv. Drug Deliver. Rev.* 59 1340–1350. 10.1016/j.addr.2007.08.012 17868951 PMC2581871

[B17] Cao X. Peng X. Zhong L. Sun R. (2014). Multiresponsive hydrogels based on xylan-type hemicelluloses and photoisomerized azobenzene copolymer as drug delivery carrier. *J. Agric. Food Chem.* 62 10000–10007. 10.1021/jf504040s 25260117

[B18] Champeau M. Thomassin J. Tassaing T. Jérôme C. (2015). Drug loading of polymer implants by supercritical CO_2_ assisted impregnation: a review. *J. Controlled Release* 209 248–259. 10.1016/j.jconrel.2015.05.002 25953410

[B19] Cidonio G. Glinka M. Kim Y. Kanczler J. M. Lanham S. A. Ahlfeld T. (2020). Nanoclay-based 3D printed scaffolds promote vascular ingrowth ex vivo and generate bone mineral tissue in vitro and in vivo. *Biofabrication* 12:035010. 10.1088/1758-5090/ab8753 32259804

[B20] Cross M. Dexter T. M. (1991). Growth factors in development, transformation, and tumorigenesis. *Cell* 64 271–280. 10.1016/0092-8674(91)90638-F 1988148

[B21] Dang H. P. Shafiee A. Lahr C. A. Dargaville T. R. Tran P. A. (2020). Local doxorubicin delivery via 3d-printed porous scaffolds reduces systemic cytotoxicity and breast cancer recurrence in mice. *Adv. Therap.* 3:2000056. 10.1002/adtp.202000056

[B22] Datta P. Barui A. Wu Y. Ozbolat V. Moncal K. K. Ozbolat I. T. (2018). Essential steps in bioprinting: from pre- to post-bioprinting. *Biotechnol. Adv.* 36 1481–1504. 10.1016/j.biotechadv.2018.06.003 29909085

[B23] Davoodi E. Sarikhani E. Montazerian H. Ahadian S. Costantini M. Swieszkowski W. (2020). Extrusion and microfluidic-based bioprinting to fabricate biomimetic tissues and organs. *Adv. Mater. Technol.* 5:1901044. 10.1002/admt.201901044 33072855 PMC7567134

[B24] de Rieux A. Ucakar B. Kaishusha Mupendwa B. P. Colau D. Feron O. Carmeliet P. (2011). 3D systems delivering VEGF to promote angiogenesis for tissue engineering. *J. Control. Release* 150 272–278. 10.1016/j.jconrel.2010.11.028 21130820

[B25] Dhammika Bandara H. M. Burdette S. C. (2012). Photoisomerization in different classes of azobenzene. *Chem. Soc. Rev.* 41 1809–1825. 10.1039/c1cs15179g 22008710

[B26] Dutta R. C. Dutta A. K. (2009). Cell-interactive 3D-scaffold; advances and applications. *Biotechnol. Adv.* 27 334–339. 10.1016/j.biotechadv.2009.02.002 19232387

[B27] Ehrbar M. Rizzi S. C. Schoenmakers R. G. San Miguel B. Hubbell J. A. Weber F. E. (2007). Biomolecular hydrogels formed and degraded via site-specific enzymatic reactions. *Biomacromolecules* 8 3000–3007. 10.1021/bm070228f 17883273

[B28] Fahimipour F. Dashtimoghadam E. Mahdi Hasani-Sadrabadi M. Vargas J. Vashaee D. Lobner D.C. (2019). Enhancing cell seeding and osteogenesis of MSCs on 3D printed scaffolds through injectable BMP2 immobilized ECM-Mimetic gel. *Dent. Mater.* 35 990–1006. 10.1016/j.dental.2019.04.004 31027908 PMC7193177

[B29] Fahimipour F. Rasoulianboroujeni M. Dashtimoghadam E. Khoshroo K. Tahriri M. Bastami F. (2017). 3D printed TCP-based scaffold incorporating VEGF-loaded PLGA microspheres for craniofacial tissue engineering. *Dent. Mater.* 33 1205–1216. 10.1016/j.dental.2017.06.016 28882369 PMC6013845

[B30] Freeman F. E. Pitacco P. van Dommelen L. H. A. Nulty J. Browe D. C. Shin J. (2020). 3D bioprinting spatiotemporally defined patterns of growth factors to tightly control tissue regeneration. *Sci. Adv.* 6:eabb5093. 10.1126/sciadv.abb5093 32851179 PMC7428335

[B31] Gao S. Tang G. Hua D. Xiong R. Han J. Jiang S. (2019). Stimuli-responsive bio-based polymeric systems and their applications. *J. Mater. Chem. B* 7 709–729. 10.1039/C8TB02491J 32254845

[B32] Gbureck U. Hölzel T. Doillon C. J. Müller F. A. Barralet J. E. (2007). Direct printing of bioceramic implants with spatially localized angiogenic factors. *Adv. Mater.* 19:795. 10.1002/adma.200601370

[B33] Geng S. Wang Y. Wang L. Kouyama T. Gotoh T. Wada S. (2017). A light-responsive self-assembly formed by a cationic azobenzene derivative and sds as a drug delivery system. *Sci. Rep.* 7:39202. 10.1038/srep39202 28051069 PMC5209711

[B34] Ghosh M. Halperin-Sternfeld M. Adler-Abramovich L. (2019). “Bio Mimicking of Extracellular Matrix,” in *Biological and Bio-Inspired Nanomaterials. Advances in Experimental Medicine and Biology*, eds Perrett S. Buell A. Knowles T. (Singapore: Springer), 371–399. 10.1007/978-981-13-9791-2_12 31713206

[B35] Gong Y. Li S. Zeng W. Yu J. Chen Y. Yu B. (2019). Controlled in vivo bone formation and vascularization using ultrasound-triggered release of recombinant vascular endothelial growth factor from poly(D,L-lactic-co-glycolicacid) microbubbles. *Front. Pharmacol.* 10:413. 10.3389/fphar.2019.00413 31068814 PMC6491501

[B36] Gonzalez-Fernandez T. Rathan S. Hobbs C. Pitacco P. Freeman F. E. Cunniffe G. M. (2019). Pore-forming bioinks to enable spatio-temporally defined gene delivery in bioprinted tissues. *J. Controlled Release* 301 13–27. 10.1016/j.jconrel.2019.03.006 30853527

[B37] Griffin D. R. Schlosser J. L. Lam S. F. Nguyen T. H. Maynard H. D. Kasko A. M. (2013). Synthesis of photodegradable macromers for conjugation and release of bioactive molecules. *Biomacromolecules* 14 1199–1207. 10.1021/bm400169d 23506440 PMC4198304

[B38] Guardado-Alvarez T. M. Sudha Devi L. Russell M. M. Schwartz B. J. Zink J. I. (2013). Activation of snap-top capped mesoporous silica nanocontainers using two near-infrared photons. *J. Am. Chem. Soc.* 135:17652. 10.1021/ja410105b PMC3862188 24015927

[B39] Guzzi E. A. Bovone G. Tibbit M. W. (2019). Universal Nanocarrier Ink Platform for Biomaterials Additive Manufacturing. *Small* 15:1905421. 10.1002/smll.201905421 31762197

[B40] Guzzi E. A. Tibbitt M. W. (2020). Additive manufacturing of precision biomaterials. *Adv. Mater.* 32:1901994. 10.1002/adma.201901994 31423679

[B41] Hacohen A. Jessel H. R. Richter-Levin A. Shefi O. (2020). Patterning of particles and live cells at single cell resolution. *Micromachines* 11:505. 10.3390/mi11050505 32429308 PMC7281171

[B42] Han Y. Lian M. Sun B. Jia B. Wu Q. Qiao Z. (2020). Preparation of high precision multilayer scaffolds based on Melt Electro-Writing to repair cartilage injury. *Theranostic* 10:10214. 10.7150/thno.47909 32929344 PMC7481411

[B43] Hao X. Silva E. A. Månsson-Broberg A. Grinnemo K. Siddiqui A. J. Dellgren G. (2007). Angiogenic effects of sequential release of VEGF-A165 and PDGF-BB with alginate hydrogels after myocardial infarction. *Cardiovasc. Res.* 75 178–185. 10.1016/j.cardiores.2007.03.028 17481597

[B44] He W. Reaume M. Hennenfent M. Lee B. P. Rajachar R. (2020). Biomimetic hydrogels with spatial- and temporalcontrolled chemical cues for tissue engineering. *Biomater. Sci.* 8 3248–3269. 10.1039/d0bm00263a 32490441 PMC7323904

[B45] Hernández A. Reyes R. Sánchez E. Rodríguez-Évora M. Delgado A. Évora C. (2012). In vivo osteogenic response to different ratios of BMP-2 and VEGF released from a biodegradable porous system. *J. Biomed. Mater. Res. Part A* 100A 2382–2319. 10.1002/jbm.a.34183 22528545

[B46] Hipfinger C. Subbiah R. Tahayeri A. Athirasala A. Horsophonphong S. Thrivikraman G. (2020). 3D printing of microgel-loaded modular LEGO-like cages as instructive scaffolds for tissue engineering. *bioRxiv* [Preprint]. 10.1101/2020.03.02.974204 32700332

[B47] Hogan K. J. Mikos A. G. (2020). Biodegradable thermoresponsive polymers: applications in drug delivery and tissue engineering. *Polymer* 211:123063. 10.1016/j.polymer.2020.123063

[B48] Hutmacher D. W. Schantz J. T. Lam C. X. F. Tan K. C. Lim T. C. (2007). State of the art and future directions of scaffold-based bone engineering from a biomaterials perspective. *J. Tssue Eng. Regen. M* 1 245–260. 10.1002/term.24 18038415

[B49] Hwa Kim D. Huegel J. Taylor B. L. Nuss C. A. Weiss S. N. Soslowsky L. J. (2020). Biocompatibility and bioactivity of an FGF-loaded microsphere-based bilayer delivery system. *Acta Biomater.* 111 341–348. 10.1016/j.actbio.2020.04.048 32428684 PMC7868956

[B50] Jacob S. Nair A. B. Patel V. Shah J. (2020). 3D printing technologies: recent development and emerging applications in various drug delivery systems. *AAPS Pharm. Sci. Tech.* 21:220. 10.1208/s12249-020-01771-4 32748243

[B51] Jelonek K. Jaworska J. Pastusiak M. Sobota M. Włodarczyk J. Karpeta-Jarzabek P. (2018). Effect of vascular scaffold composition on release of sirolimus. *Eur. J. Pharm. Biopharm.* 132 41–49. 10.1016/j.ejpb.2018.08.015 30179737

[B52] Jiang Y. Yang Y. Zheng X. Yi Y. Chen X. Li Y. (2020). Multifunctional load-bearing hybrid hydrogel with combined drug release and photothermal conversion functions. *NPG Asia Mater.* 12:18. 10.1038/s41427-020-0199-6

[B53] Kang Y. Datta P. Shanmughapriya S. Ozbolat I. T. (2020). 3D Bioprinting of tumor models for cancer research. *ACS Appl. Bio Mater.* 3 5552–5573. 10.1021/acsabm.0c00791 35021789

[B54] Katt M. E. Placone A. L. Wong A. D. Xu Z. S. Searson P. C. (2016). In vitro tumor models: advantages, disadvantages, variables, and selecting the right platform. *Front. Bioeng. Biotechnol.* 4:12. 10.3389/fbioe.2016.00012 26904541 PMC4751256

[B55] Kelly D. C. Raftery R. M. Curtin C. M. O’Driscoll C. M. O’Brien F. J. (2019). Scaffold-based delivery of nucleic acid therapeutics for enhancedbone and cartilage repair. *J. Orthop. Res.* 37:1671. 10.1002/jor.24321 31042304

[B56] Kim H. Park H. Lee J. W. Lee K. Y. (2016). Magnetic field-responsive release of transforming growth factor beta 1 from heparin-modified alginate ferrogels. *Carbohyd. Polym.* 151:467. 10.1016/j.carbpol.2016.05.090 27474590

[B57] Koons G. L. Mikos A. G. (2019). Progress in three-dimensional printing with growth factors. *J. Controlled Release* 295:50. 10.1016/j.jconrel.2018.12.035 30579982 PMC6358495

[B58] Li J. Mooney D. J. (2016). Designing hydrogels for controlled drug delivery. *Nat. Rev. Mater.* 1:16071. 10.1038/natrevmats.2016.71 29657852 PMC5898614

[B59] Li X. Wang Y. Wang Z. Qi Y. Li L. Zhang P. (2018). Composite PLA/PEG/nHA/dexamethasone scaffold prepared by 3D printing for bone regeneration. *Macromol. Biosci.* 18:1800068. 10.1002/mabi.201800068 29687630

[B60] Lin C. Y. J. Kang H. Hollister S. J. (2020). “Biomechanics of osteo-synthetics,” in *Frontiers in Orthopaedic Biomechanics*, eds Cheng C. K. Woo S. Y. (Singapore: Springer), 397–425. 10.1007/978-981-15-3159-0_15

[B61] Linsley C. S. Wu B. M. (2017). Recent advances in light-responsive ondemand drug-delivery systems. *Ther. Deliv.* 8 89–107. 10.4155/tde-2016-0060 28088880 PMC5561969

[B62] Liu H. Du Y. Yang G. Hu X. Wang L. Liu B. (2020). Delivering proangiogenic factors from 3D-printed polycaprolactone scaffolds for vascularized bone regeneration. *Adv. Healthc. Mater.* 9:2000727. 10.1002/adhm.202000727 32743958

[B63] Liu Y. Yu H. Liu Y. Liang G. Zhang T. Hu Q. (2016). Dual drug spatiotemporal release from functional gradient scaffolds prepared using 3D Bioprinting and Electrospinning. *Polym. Eng. Sci.* 56 170–177. 10.1002/pen.24239

[B64] Loebel C. Burdick J. A. (2018). Engineering stem and stromal cell therapiesfor musculoskeletal tissue repair. *Cell Stem Cell* 22 325–339. 10.1016/j.stem.2018.01.014 29429944 PMC5834383

[B65] Lu T. Ten Hagen T. L. M. (2020). A novel kinetic model to describe the ultra-fast triggered release of thermosensitive liposomal drug delivery systems. *J. Controlled Release* 324 669–678. 10.1016/j.jconrel.2020.05.047 32512013

[B66] Lu X. Jin H. Quesada C. Farrell E. C. Huang L. Aliabouzar M. (2020). Spatially-directed cell migration in acoustically-responsive scaffolds through the controlled delivery of basic fibroblast growth factor. *Acta Biomater.* 113 217–227. 10.1016/j.actbio.2020.06.015 32553916 PMC7423759

[B67] Mao S. Pang Y. Liu T. Shao Y. He J. Yang H. (2020). Bioprinting of in vitro tumor models for personalized cancer treatment: a review. *Biofabrication* 12:042001. 10.1088/1758-5090/ab97c0 32470967

[B68] Marizza P. Keller S. S. Müllertz A. Boisen A. (2014). Polymer-filled microcontainers for oral delivery loaded using supercritical impregnation. *J. Control. Release* 173 1–9. 10.1016/j.jconrel.2013.09.022 24096018

[B69] McHugh K. J. Nguyen T. D. Linehan A. R. Yang D. Behrens A. M. Rose S. (2017). Fabrication of fillable microparticles and other complex 3D microstructures. *Science* 359 1138–1142. 10.1126/science.aaf7447 28912242 PMC6510330

[B70] Mekhileri N. V. Lim K. S. Brown G. C. J. Mutreja I. Schon B. S. Hooper G. J. (2018). Automated 3D bioassembly of micro-tissues for biofabrication of hybrid tissue engineered constructs. *Biofabrication* 10:024103. 10.1088/1758-5090/aa9ef1 29199637

[B71] Mirza I. Saha S. (2020). Biocompatible anisotropic polymeric particles: synthesis, characterization, and biomedical applications. *ACS Appl. Biol. Mater.* 3:8241. 10.1021/acsabm.0c01075 35019601

[B72] Mohammed A. Elshaer A. Sareh P. Elsayed M. Hassanin H. (2020). Additive manufacturing technologies for drug delivery applications. *Int. J. Pharm.* 580:119245. 10.1016/j.ijpharm.2020.119245 32201252

[B73] Moncion A. Lin M. O‘Neill E. G. Franceschi R. T. Kripfgans O. D. Putnam A. J. (2017). Controlled release of basic fibroblast growth factor for angiogenesis using acoustically-responsive scaffolds. *Biomaterials* 140 26–36. 10.1016/j.biomaterials.2017.06.012 28624705 PMC5537721

[B74] Mondal S. Das S. Nandi A. K. (2020). A review on recent advances in polymer and peptide hydrogels. *Soft Matter.* 16 1404–1454. 10.1039/c9sm02127b 31984400

[B75] Moreira A. Carneiro J. Campos J. B. L. M. Miranda J. M. (2021). Production of hydrogel microparticles in microfluidic devices: a review. *Microfluid. Nanofluid.* 25:10. 10.1007/s10404-020-02413-8

[B76] Moreno Madrid A. P. Mariel Vrecha S. Sanchez M. A. Rodriguez A. P. (2019). Advances in additive manufacturing for bone tissue engineering scaffolds. *Mat. Sci. Eng. C* 100:631. 10.1016/j.msec.2019.03.037 30948100

[B77] Murphy C. Kolan K. Li W. Semon J. Day D. Leu M. (2017). 3D bioprinting of stem cells and polymer/bioactive glass composite scaffolds for bone tissue engineering. *Int. J. Bioprinting* 3:54. 10.18063/IJB.2017.01.005 33094180 PMC7575634

[B78] Ngo T. T. Hoffman L. Hoople G. D. Trevena W. Shakya U. Barr G. (2020). Surface morphology and drug loading characterization of 3D-printedmethacrylate-based polymer facilitated by supercritical carbondioxide. *J. Supercrit. Fluid* 160:104786. 10.1016/j.supflu.2020.104786

[B79] Oliva N. Almquist B. D. (2020). Spatiotemporal delivery of bioactive molecules for wound healing using stimuli-responsive biomaterials. *Adv. Drug Delivery Rev.* 161–162 22–41. 10.1016/j.addr.2020.07.021 32745497

[B80] Omidi M. Almeida L. Tayebi L. (2020). Microfluidic-assisted fabrication of reverse micelle/PLGA hybrid microspheres for sustained vascular endothelial growth factor delivery. *Biotechnol. Appl. Biochem.* (inpress). 10.1002/bab.1971 32533571

[B81] Oztan Y. C. Nawafleh N. Zhou Y. Liyanage P. Y. Hettiarachchi S. D. Seven E. S. (2020). Recent advances on utilization of bioprinting for tumor modelling. *Bioprinting* 18:e00079. 10.1016/j.bprint.2020.e00079 32099931 PMC7041912

[B82] Pal A. Smith C. I. Palade J. Nagaraju S. Alarcon-Benedetto B. A. Kilbourne J. (2020). Poly(N-isopropylacrylamide)-based dual-crosslinking biohybrid injectable hydrogels for vascularization. *Acta Biomater.* 107 138–151. 10.1016/j.actbio.2020.02.041 32126310

[B83] Palmese L. L. Fan M. Scott R. A. Tan H. Kiick K. L. (2020). Multi-stimuli-responsive, liposome-crosslinked poly(ethylene glycol) hydrogels for drug delivery. *J. Biomat. Sci. Polym E* 32 635–656. 10.1080/09205063.2020.1855392 33231137 PMC8659393

[B84] Parak A. Pradeep P. du Toit L. C. Kumar P. Choonara Y. E. Pillay V. (2019). Functionalizing bioinks for 3D bioprinting applications. *Drug Discov. Today* 24 198–205. 10.1016/j.drudis.2018.09.012 30244080

[B85] Pedersen S. L. Huynh T. H. Pöschko P. Fruergaard A. S. Olesen M. T. J. Chen Y. (2020). Remotely triggered liquefaction of hydrogel materials. *ACS Nano.* 14 9145–9155. 10.1021/acsnano.0c04522 32615036

[B86] Peng Z. Sun H. Bunpetch V. Koh Y. Wen Y. Wu D. (2021). The regulation of cartilage extracellular matrix homeostasis in joint cartilage degeneration and regeneration. *Biomaterials* 268:120555. 10.1016/j.biomaterials.2020.120555 33285440

[B87] Poldervaart M. T. Gremmels H. van Deventer K. Fledderus J. O. Cumur Öner F. Verhaar M. C. (2014). Prolonged presence of VEGF promotes vascularization in 3D bioprinted scaffolds with defined architecture. *J. Control. Release* 184 58–66. 10.1016/j.jconrel.2014.04.007 24727077

[B88] Radhakrishnan J. Varadaraji S. Kumar Dash S. Sharma A. Shanker Verma R. (2020). Organotypic cancer tissue models for drug screening: 3D constructs, bioprinting and microfluidic chips. *Drug Discov. Today* 25 879–890. 10.1016/j.drudis.2020.03.002 32165322

[B89] Raimondo T. M. Li H. Kwee B. J. Kinsley S. Budina E. Andersona E. M. (2019). Combined delivery of VEGF and IGF-1 promotes functional innervation in mice and improves muscle transplantation in rabbits. *Biomaterials* 216:119246. 10.1016/j.biomaterials.2019.119246 31203034

[B90] Randall C. L. Leong T. G. Bassik N. Gracias D. H. (2007). 3D lithographically fabricated nanoliter containers for drug delivery. *Adv. Drug Deliver. Rev.* 59 1547–1561. 10.1016/j.addr.2007.08.024 17919768

[B91] Richard C. Neild A. Cadarso V. J. (2020). The emerging role of microfluidics in multimaterial 3D bioprinting. *Lab. Chip.* 20 2044–2056. 10.1039/c9lc01184f 32459222

[B92] Richardson T. P. Peters M. C. Ennet A. B. Mooney D. J. (2001). Polymeric system for dual growth factor delivery. *Nat. Biotechnol.* 19 1029–1034. 10.1038/nbt1101-1029 11689847

[B93] Ruskowitz E. R. DeForest C. A. (2018). Photoresponsive biomaterials for targeted drug delivery and 4D cell culture. *Nat. Rev. Mater.* 3:17087. 10.1038/natrevmats.2017.87

[B94] Salerno A. Cesarelli G. Pedram P. Netti P. A. (2019). Modular strategies to build cell-free and cell-laden scaffolds towards bioengineered tissues and organs. *J. Clin. Med.* 8:1816. 10.3390/jcm8111816 31683796 PMC6912533

[B95] Salerno A. Diéguez S. Diaz-Gomez L. Gómez-Amoza J. L. Magariños B. Concheiro A. (2017). Synthetic scaffolds with full pore interconnectivity for bone regeneration prepared by supercritical foaming using advanced biofunctional plasticizers. *Biofabrication* 9:035002. 10.1088/1758-5090/aa78c5 28604361

[B96] Salerno A. Domingo C. (2015). Bio-based polymers, supercritical fluids and tissue engineering. *Process Biochem.* 50 826–838. 10.1016/j.procbio.2015.02.009

[B97] Salerno A. Fernández-Gutiérrez M. San Román del Barrio J. Domingo Pascual C. (2014). Macroporous and nanometre scale fibrous PLA and PLA–HA composite scaffolds fabricated by a bio safe strategy. *RSC Adv.* 4:61491. 10.1039/C4RA07732F

[B98] Salerno A. Levato R. Mateos-Timoneda M. A. Engel E. Netti P. A. Planell J. (2013). Modular polylactic acid microparticle-based scaffolds prepared via microfluidic emulsion/solvent displacement process: fabrication, characterization, and in vitro mesenchymal stem cells interaction study. *J. Biomed. Mater. Res. Part A* 101A 720–732. 10.1002/jbm.a.34374 22941938

[B99] Salerno A. Netti P. A. (2014). “Introduction to biomedical foams,” in *Biomedical Foams for Tissue Engineering Applications*, ed. Netti P. A. (Amsterdam: Woodhead Publishing), 3–39.

[B100] Salerno A. Saurina J. Domingo C. (2015). Supercritical CO2 foamed polycaprolactone scaffolds for controlled delivery of 5-fluorouracil, nicotinamide and triflusal. *Int. J. Pharm.* 496 654–663. 10.1016/j.ijpharm.2015.11.012 26570986

[B101] Salerno A. Verdolotti L. Raucci M. G. Saurina J. Domingo C. Lamanna R. (2018). Hybrid gelatin-based porous materials with a tunable multiscale morphology for tissue engineering and drug delivery. *Eur. Polym. J.* 99 230–239. 10.1016/j.eurpolymj.2017.12.024

[B102] Santos M. I. Reis R. L. (2020). Vascularization in bone tissue engineering: physiology, current strategies, major hurdles and future challenges. *Macromol. Biosci.* 10 12–27. 10.1002/mabi.200900107 19688722

[B103] Saraf S. Jain A. Tiwari A. Verma A. Kumar Panda P. Jain S. K. (2020). Advances in liposomal drug delivery to cancer: an overview. *J. Drug. Deliv. Sci. Tech.* 56:101549. 10.1016/j.jddst.2020.101549

[B104] Saraswat Y. C. Ibis F. Rossi L. Sasso L. Eral H. B. Fanzio P. (2020). Shape anisotropic colloidal particle fabrication using 2-photon polymerization. *J. Colloid Interf. Sci.* 564 43–51. 10.1016/j.jcis.2019.12.035 31901833

[B105] Sarkar N. Bose S. (2020). Controlled release of soy isoflavones from multifunctional 3D printed bone tissue engineering scaffolds. *Acta Biomater.* 114 407–420. 10.1016/j.actbio.2020.07.006 32652224 PMC8009492

[B106] Saska S. Pires L. C. Cominotte M. A. Mendes L. S. de Oliverira M. F. Maia I. A. (2018). Three-dimensional printing and in vitro evaluation of poly(3-hydroxybutyrate) scaffolds functionalized with osteogenic growth peptide for tissue engineering. *Mater. Sci. Eng. C* 89:265. 10.1016/j.msec.2018.04.016 29752098

[B107] Shafiee A. (2020). Design and fabrication of three-dimensional printed scaffolds for cancer precision medicine. *Tissue Eng. Part A* 26 305–317. 10.1089/ten.tea.2019.0278 31992154

[B108] Shi X. Cheng Y. Wang J. Chen H. Wang X. Li X. (2020). 3D printed intelligent scaffold prevents recurrence and distal metastasis of breast cancer. *Theranostics* 10 10652–10664. 10.7150/thno.47933 32929372 PMC7482818

[B109] Shrike Zhang Y. Duchamp M. Oklu R. Ellisen L. W. Langer R. Khademhosseini A. (2016). Bioprinting the Cancer Microenvironment. *ACS Biomater. Sci. Eng.* 2 1710–1721. 10.1021/acsbiomaterials.6b00246 28251176 PMC5328669

[B110] Shvartsman D. Storrie-White H. Lee K. Kearney C. Brudno Y. Ho N. (2014). Sustained delivery of VEGF maintains innervation and promotes reperfusion in ischemic skeletal muscles via NGF/GDNF signaling. *Mol. Ther.* 22 1243–1253. 10.1038/mt.2014.76 24769910 PMC4089004

[B111] Shultz R. B. Zhong Y. (2021). Hydrogel-based local drug delivery strategies for spinal cord repair. *Neural Regen. Res.* 16 247–253. 10.4103/1673-5374.290882 32859771 PMC7896229

[B112] Sigaux N. Pourchet L. Breton P. Brosset S. Louvrier A. Marquette C. A. (2019). 3D Bioprinting:principles, fantasies and prospects. *J. Stomatol. Oral Maxillofac Surg.* 120 128–132. 10.1016/j.jormas.2018.12.014 30609384

[B113] Silva E. D. Babo P. S. Costa-Almeida R. Domingues R. M. A. Mendes B. B. Paz E. (2018). Multifunctional magnetic-responsive hydrogels to engineer tendon-to-bone interface. *Nanomed. Nanotechnol.* 14 2375–2385. 10.1016/j.nano.2017.06.002 28614734

[B114] Skardal A. Murphy S. V. Crowell K. Mack D. Atala A. Soker S. (2017). A tunable hydrogel system for long-term release of cell-secreted cytokines and bioprinted in situ wound cell delivery. *J. Biomed. Mater. Res. Part B Appl. Biomater.* 105B:1986. 10.1002/jbm.b.33736 27351939 PMC9159053

[B115] Steele J. A. M. McCullen S. D. Callanan D. Autefage H. Accardi M. A. Dini D. (2014). Combinatorial scaffold morphologies for zonal articular cartilage engineering. *Acta Biomater.* 10 2065–2075. 10.1016/j.actbio.2013.12.030 24370641 PMC3991416

[B116] Stewart S. A. Domínguez-Robles J. MvIlorum V. J. Gonzalez Z. Utomo E. Mancuso E. (2020). Poly(caprolactone)-based coatings on 3D-printed biodegradable implants: a novel strategy to prolong delivery of hydrophilic drugs. *Mol. Pharm.* 17 3487–3500. 10.1021/acs.molpharmaceut.0c00515 32672976 PMC7482401

[B117] Stubbe B. G. De Smedt S. C. Demeester J. (2004). “Programmed Polymeric Devices” for pulsed drug delivery. *Pharm. Res.* 21 1732–1740. 10.1023/B:PHAM.0000045223.45400.01 15553216

[B118] Sun B. Lian M. Han Y. Mo W. Jiang W. Qiao Z. (2021). A 3D-Bioprinted dual growth factor-releasing intervertebral disc scaffold induces nucleus pulposus and annulus fibrosus reconstruction. *Bioact. Mater.* 6 179–190. 10.1016/j.bioactmat.2020.06.022 32913927 PMC7451922

[B119] Sun Q. Chen R. R. Shen Y. Mooney D. J. Rajagopalan S. Grossman P. M. (2005). Sustained vascular endothelial growth factor delivery enhances angiogenesis and perfusion in ischemic hind limb. *Pharm. Res.* 22 1110–1116. 10.1007/s11095-005-5644-2 16028011

[B120] Sun Y. You Y. Jiang W. Wang B. Wu Q. Dai K. (2020a). 3D bioprinting dual-factor releasing and gradient-structured constructs ready to implant for anisotropic cartilage regeneration. *Sci. Adv.* 6:eaay1422. 10.1126/sciadv.aay1422 32917692 PMC11206535

[B121] Sun Y. You Y. Jiang W. Wu Q. Wang B. Dai K. (2020b). Generating ready-to-implant anisotropic menisci by 3D-bioprintingprotein-releasing cell-laden hydrogel-polymer composite scaffold. *Appl. Mater. Today* 18:100469. 10.1016/j.apmt.2019.100469

[B122] Tamjid E. Bohlouli M. Mohammadi S. Alipour H. Nikkhah M. (2020). Sustainable drug release from highly porous and architecturally engineered composite scaffolds prepared by 3D printing. *J. Biomed. Mater. Res. Part A* 108:1426. 10.1002/jbm.a.36914 32134569

[B123] Tao J. Zhang Y. Shen A. Diao L. Wang L. Cai D. (2020). Injectable chitosan-based thermosensitive hydrogel/nanoparticle-loaded system for local delivery of vancomycin in the treatment of osteomyelitis. *Int. J. Nanomed.* 15 5855–5871. 10.2147/IJN.S247088 32848394 PMC7428380

[B124] Tarafder S. Koch A. Jun Y. Chou C. Awadallah M. R. Lee C. H. (2016). Micro-precise spatiotemporal delivery system embedded in 3D printing for complex tissue regeneration. *Biofabrication* 8:025003. 10.1088/1758-5090/8/2/025003 27108484

[B125] Thébault C. J. Ramniceanu G. Boumati S. Michel A. Seguin J. Larrat B. (2020). Theranostic MRI liposomes for magnetic targeting and ultrasound triggered release of the antivascular CA4P. *J. Controlled Release* 322 137–148. 10.1016/j.jconrel.2020.03.003 32145266

[B126] Tomeh M. A. Zhao X. (2020). Recent advances in microfluidics for the preparation of drug and gene delivery systems. *Mol. Pharm.* 17 4421–4434. 10.1021/acs.molpharmaceut.0c00913 33213144

[B127] Visscher L. E. Phuc Dang H. Knackstedt M. A. Hutmacher D. W. Tran P. A. (2018). 3D printed Polycaprolactone scaffolds with dual macro-microporosity for applications in local delivery of antibiotics. *Mat. Sci. Eng. C* 87 78–89. 10.1016/j.msec.2018.02.008 29549952

[B128] Wagner E. R. Parry J. Dadsetan M. Bravo D. Riester S. M. Van Wijnen A. J. (2018). VEGF-mediated angiogenesis and vascularization of a fumarate-crosslinked polycaprolactone (PCLF) scaffold. *Connect. Tissue Res.* 59 542–549. 10.1080/03008207.2018.1424145 29513041

[B129] Wang C. Ye X. Zhao Y. Bai L. He Z. Tong Q. (2020). Cryogenic 3D printing of porous scaffolds for in situ delivery of 2D black phosphorus nanosheets, doxorubicin hydrochloride and osteogenic peptide for treating tumor resection-induced bone defects. *Biofabrication* 12:035004. 10.1088/1758-5090/ab6d35 31952065

[B130] Wang H. Zhang W. Gao C. (2015). Shape Transformation of light-responsive pyrene-containing micelles and their influence on cytoviability. *Biomacromolecules* 16 2276–2281. 10.1021/acs.biomac.5b00497 26133965

[B131] Wang L. Deng F. Wang W. Li A. Lu C. Chen H. (2018). Construction of injectable self-healing macroporous hydrogels via a template-free method for tissue engineering and drug delivery. *ACS Appl. Mater. Interf.* 10 36721–36732. 10.1021/acsami.8b13077 30261143

[B132] Wang W. Sun L. Zhang P. Song J. Liu W. (2014). An anti-inflammatory cell-free collagen/resveratrol scaffold for repairing osteochondral defects in rabbits. *Acta Biomater.* 10:4983. 10.1016/j.actbio.2014.08.022 25169257

[B133] Wang X. Wenk E. Zhang X. Meinel L. Vunjak-Novakovic G. Kaplan D. L. (2009). Growth factor gradients via microsphere delivery in biopolymer scaffolds for osteochondral tissue engineering. *J. Control. Release* 134 81–90. 10.1016/j.jconrel.2008.10.021 19071168 PMC2698962

[B134] Wen Y. Dai N. Hsu S. (2019). Biodegradable water-based polyurethane scaffolds with a sequential release function for cell-free cartilage tissue engineering. *Acta Biomater.* 88 301–313. 10.1016/j.actbio.2019.02.044 30825604

[B135] Xiong S. Zhang X. Lu P. Wu Y. Wang Q. Sun H. (2017). A Gelatin-sulfonated Silk Composite Scaffold based on 3D printing technology enhances skin regeneration by stimulating epidermal growth and dermal neovascularization. *Sci. Rep.* 7:4288. 10.1038/s41598-017-04149-y 28655891 PMC5487355

[B136] Yang Y. Qiao X. Huang R. Chen H. Shi X. Wang J. (2020). E-jet 3D printed drug delivery implants to inhibit growth and metastasis of orthotopic breast cancer. *Biomaterials* 230:119618. 10.1016/j.biomaterials.2019.119618 31757530

[B137] Yao Q. Nooeaid P. Roether J. A. Dong Y. Zhang Q. Boccaccini A. R. (2013). Bioglass^®^ -based scaffolds incorporating polycaprolactone and chitosan coatings for controlled vancomycin delivery. *Ceram. Int.* 39 7517–7522. 10.1016/j.ceramint.2013.03.002

[B138] Ye L. Wang J. Liao C. Li S. Fang Y. Yang Z. (2019). 3D printed composite scaffolds incorporating ruthenium complex–loaded liposomes as a delivery system to prevent the proliferation of MG-63 cells. *Macromol. Mater. Eng.* 304:1900295.

[B139] Yuba E. (2020). Development of functional liposomes by modification of stimuli-responsive materials and their biomedical applications. *J. Mater. Chem.* 8 1093–1107. 10.1039/C9TB02470K 31960007

[B140] Zhang H. Gong W. Wang Z. Yuan S. Xie X. Yang Y. (2014). Preparation, characterization, and pharmacodynamics of thermosensitive liposomes containing Docetaxel. *J. Pharm. Sci.* 103 2177–2183. 10.1002/jps.24019 24846075

[B141] Zhang Y. Zhao J. Yang G. Zhou Y. Gao W. Wu G. (2019). Sustainable drug release from highly porous and architecturally engineered composite scaffolds prepared by 3D printing. *J. Biomatr. Sci. Polym. E* 30 547–560. 10.1080/09205063.2019.1586303 30897033

[B142] Zhao W. Li Y. Zhang X. Zhang R. Hu Y. Boyer C. (2020). Photo-responsive supramolecular hyaluronic acid hydrogels for accelerated wound healing. *J. Controlled Release* 323 24–35. 10.1016/j.jconrel.2020.04.014 32283209

[B143] Zhou C. Yang K. Wang K. Pei X. Dong Z. Hong Y. (2016). Combination of fused deposition modeling and gas foaming technique to fabricated hierarchical macro/microporous polymer scaffolds. *Mater. Design.* 109:415. 10.1016/j.matdes.2016.07.094

[B144] Zhu S. Chen P. Chen Y. Li M. Chen C. Lu H. (2020). 3D-Printed Extracellular Matrix/Polyethylene glycol diacrylate hydrogel incorporating the anti-inflammatory phytomolecule honokiol for regeneration of osteochondral defects. *Am. J. Sport Med.* 48 2808–2818. 10.1177/0363546520941842 32762553

[B145] Zhu W. Cui H. Boualam B. Masood F. Flynn E. Rao R. D. (2018). 3D bioprinting mesenchymal stem cell-laden construct with core–shell nanospheres for cartilage tissue engineering. *Nanotechnology.* 29:185101. 10.1088/1361-6528/aaafa1 29446757

[B146] Zhu W. Holmes B. Glazer R. I. Zhang L. G. (2016). 3D printed nanocomposite matrix for the study of breast cancer bone metastasis. *Nanomed. Nanotechnol.* 12 69–79. 10.1016/j.nano.2015.09.010 26472048

